# Re‐Entrant Super‐Repellent Metallic Structures for Robust Anti‐Icing

**DOI:** 10.1002/advs.202508272

**Published:** 2025-08-19

**Authors:** Daizhou Li, Lizhong Wang, Rui Peng, Ziyan Song, Zhao Liu, Zhixuan Chang, Hongjun Zhang, Peixun Fan, Minlin Zhong

**Affiliations:** ^1^ Laser Materials Processing Research Center Key Laboratory for Advanced Materials Processing Technology (Ministry of Education) Joint Research Center for Advanced Materials & Anti‐icing of Tsinghua University (SMSE)‐AVIC ARI School of Materials Science and Engineering Tsinghua University Beijing 100084 P. R. China

**Keywords:** anti‐icing/deicing performance, cassie–baxter stability, laser micro/nanofabrication, re‐entrant structure, super‐repellent surface

## Abstract

Superhydrophobic surfaces exhibit significant potential for anti‐icing applications. However, sustaining the superhydrophobic Cassie‐Baxter state under conditions of low temperature, high humidity, and dynamic impacts remains a critical challenge. Re‐entrant structures, as a milestone advance in the study of super‐repellent surfaces, not only render material surfaces the Cassie‐Baxter state when contacting with liquids beyond water, but also raise a capability in keeping the super‐repellent surfaces effective under broader environmental conditions. However, most studies on re‐entrant super‐repellent structures focus on their room‐temperature performances. The water‐repellent capabilities of re‐entrant structures under low‐temperature conditions and their anti‐icing performances, in particular, are yet to be elucidated. Herein, re‐entrant structures are designed and fabricated directly on metal surfaces, which exhibit remarkable anti‐icing performances, including ultra‐long icing delay (> 600 min), ultra‐low ice adhesion strength (1.6 kPa), and significant durability (ice adhesion strength staying < 25 kPa after 100 deicing cycles). Through systematic characterizations and careful thermodynamic analysis, the underlying mechanisms for the remarkable anti‐icing performances are revealed. This study not only validates the effectiveness of re‐entrant structures under low‐temperature conditions but also engineers the design and fabrication of re‐entrant structures for excellent anti‐icing performances, establishing a new paradigm for designing robust anti‐icing surfaces.

## Graphical abstract

Breakthrough re‐entrant super‐repellent structures engineered on metals demonstrate unprecedented anti‐icing performances: >600 min icing delay, 1.6 kPa ultralow ice adhesion strength, and 100‐cycle deicing durability. Thermodynamic analysis elucidates novel anti‐icing mechanisms and underlying design principles for exceptional ice‐resistance under harsh environments, establishing a new paradigm to construct robust anti‐icing surfaces.

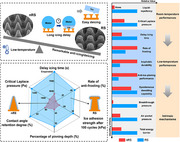

## 1 Introduction

The past three decades have witnessed the flourishing of studies on superhydrophobic surfaces.^[^ ^1^, ^2^ ^]^ However, efforts to seek practical applications of superhydrophobic surfaces (e.g., for self‐cleaning,^[^ ^3^ ^]^ water harvesting,^[^ ^4^ ^]^ anti‐icing,^[^ ^5^, ^6^ ^]^ anti‐corrosion,^[^ ^7^ ^]^ etc.) have been frustrated by common and major challenges in keeping superhydrophobic surfaces effective (particularly, staying in the Cassie‐Baxter (CB) state) under complicated and even extreme real conditions.^[^ ^8^, ^9^ ^]^ Anti‐icing is a typical application requiring techniques capable of performing under extreme conditions.^[^ ^10^, ^11^, ^12^ ^]^ As one of the most promising passive anti‐icing strategies, significant achievements have been made on superhydrophobic anti‐icing surfaces, particularly in inhibiting ice nucleation, delaying droplet freezing, and reducing ice adhesion.^[^ ^13^, ^14^, ^15^ ^]^ However, the demonstrations on superhydrophobic anti‐icing surfaces were primarily conducted in static and mild icing conditions.^[^ ^16^, ^17^, ^18^ ^]^ Striking efforts have been devoted to enhancing the stability and durability of superhydrophobic surfaces to support their practical anti‐icing applications in critical fields, e.g., on aircraft.^[^ ^19^, ^20^, ^21^ ^]^

**Figure 1:**
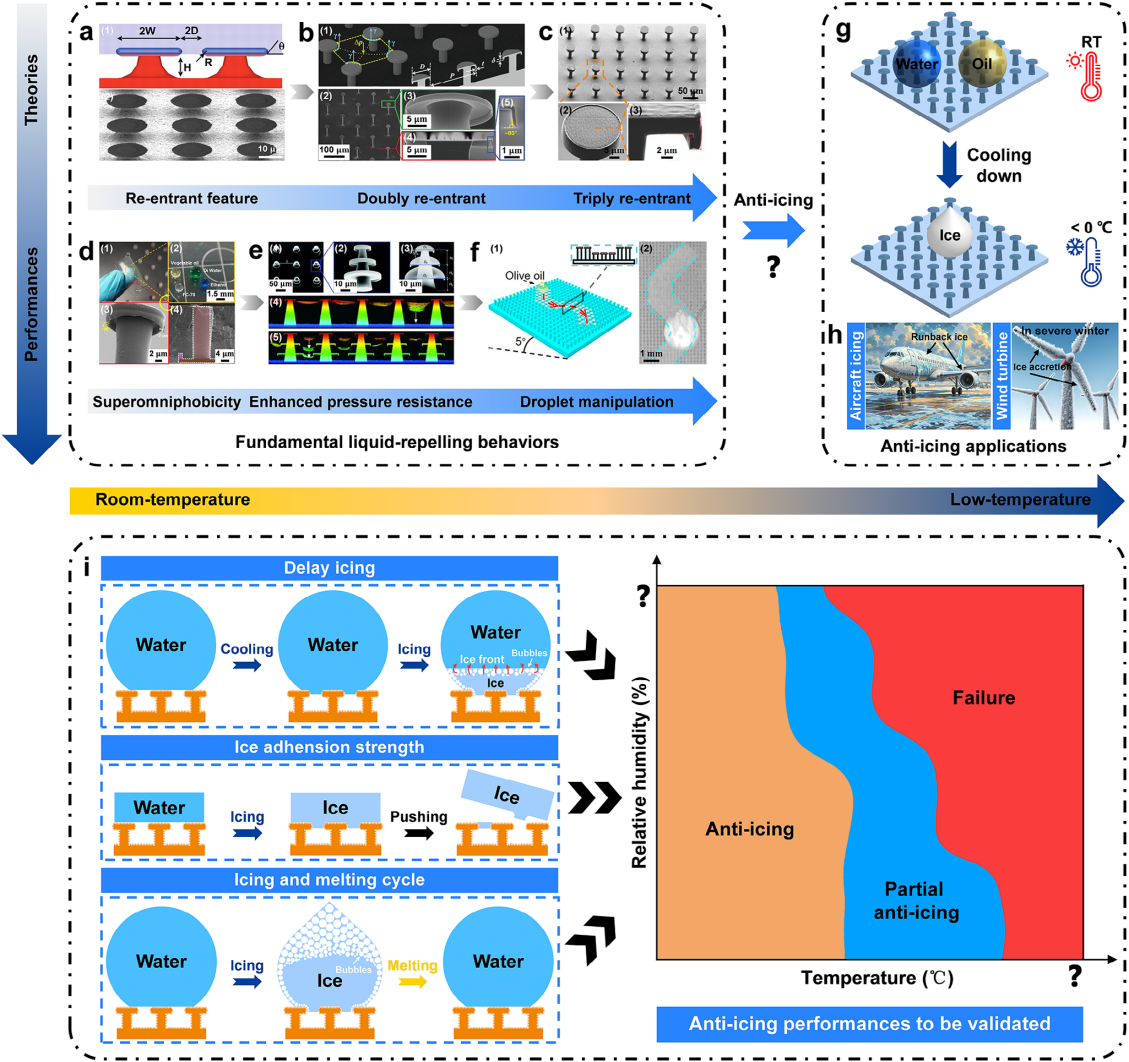
A brief technology roadmap for the studies of RSs. The theoretical studies and design of RSs have spanned over a) Singly RS, reproduced with permission.^[^ ^22^ ^]^ Copyright 2007, American Association for the Advancement of Science; b) Doubly RS, reproduced with permission.^[^ ^27^ ^]^ Copyright 2014, American Association for the Advancement of Science; and c) Triply RS, reproduced/adapted with permission.^[^ ^28^ ^]^ Copyright 2018, Wiley. The performances studied on RSs include d) Superomniphobicicy, reproduced with permission.^[^ ^35^ ^]^ Copyright 2022, American Chemical Society; e) Enhanced pressure resistance, reproduced with permission.^[^ ^32^ ^]^ Copyright 2021, American Institute of Physics; and f) Droplet manipulation, reproduced with permission.^[^ ^36^ ^]^ Copyright 2021, American Chemical Society. g,h) However, how the RSs will perform under low‐temperature conditions (particularly, whether they can be used for various anti‐icing applications) remains an open question. i) Anti‐icing tests to be presented in the following sections: delay icing, ice adhesion strength, icing and melting cycle, and the effectiveness of anti‐icing.

Re‐entrant structures (RSs), since their first demonstrations, have boosted remarkable advances in the studies of superhydrophobic surfaces.^[^ ^22^, ^23^, ^24^ ^]^ The significance of RSs in enhancing the liquid‐repellent properties of superhydrophobic surfaces has been well endorsed. In addition to expanding the repellency of superhydrophobic surfaces from water to liquids of quite low surface tensions (e.g., decane and octane^[^ ^22^ ^]^), RSs have also enhanced the stability of superhydrophobic surfaces under extreme conditions, including high weber number impact by low‐surface‐tension droplets.^[^ ^25^, ^26^ ^]^ The prominent uniqueness of RSs has driven their design evolution from simple to hierarchical ones, e.g., doubly and triply RSs,^[^ ^27^, ^28^ ^]^ branched RSs,^[^ ^29^ ^]^ as well as various structures with re‐entrant geometrical features, although not strictly designed (**Figure** **1**a–c).^[^ ^23^ ^]^

Conventional superhydrophobic surfaces enable superhydrophobicity primarily by minimizing the solid fractions (or solid‐water contact areas) on material surfaces and usually require micro/nano structures with sharp geometries (e.g., microcones,^[^ ^5^ ^]^ nanotubes,^[^ ^30^ ^]^ nanowires (NWs),^[^ ^31^ ^]^ etc.), which are relatively fragile and vulnerable to various external damages. In contrast, the re‐entrant superhydrophobic structures rely on their unique re‐entrant and T‐shaped designs, which can not only enhance the CB state stability but also allow more solid fraction on material surfaces and hence, elevated durability.^[^ ^32^, ^33^, ^34^ ^]^ However, major research interests on superhydrophobic RSs to date are devoted on their fundamental liquid‐repellent and droplet‐manipulation behaviors^[^ ^35^, ^36^ ^]^ particularly under room‐temperature conditions (Figure 1d–f). The water‐repellent capabilities of re‐entrant superhydrophobic structures under low‐temperature conditions and, correspondingly, their anti‐icing performances are yet to be elucidated (Figure 1g,h), mainly due to limited technical routines to their facile fabrication on engineering materials, e.g., metals.^[^ ^24^ ^]^

In this study, we designed and fabricated RSs directly on metal surfaces in an engineering manner, based on which a comprehensive assessment on the anti‐icing performances (including delay icing, reduced ice‐adhesion strength, and cycled icing‐melting behaviors, as schematically illustrated in Figure 1i) of RSs was conducted. The results demonstrate that the RSs developed exhibit remarkably enhanced anti‐icing performances and robust CB state stability, ensuring long icing delay, facile ice removal, and reusable icephobicity. Through sketching out the anti‐icing performances of superhydrophobic RSs, a fundamental understanding on whether RSs can be used for anti‐icing and the essential regimes for RSs to benefit anti‐icing can be established.

## 2 Results and Discussion

### 2.1 Super‐Repellency and CB Stability of the RSs Fabricated

**Figure 2:**
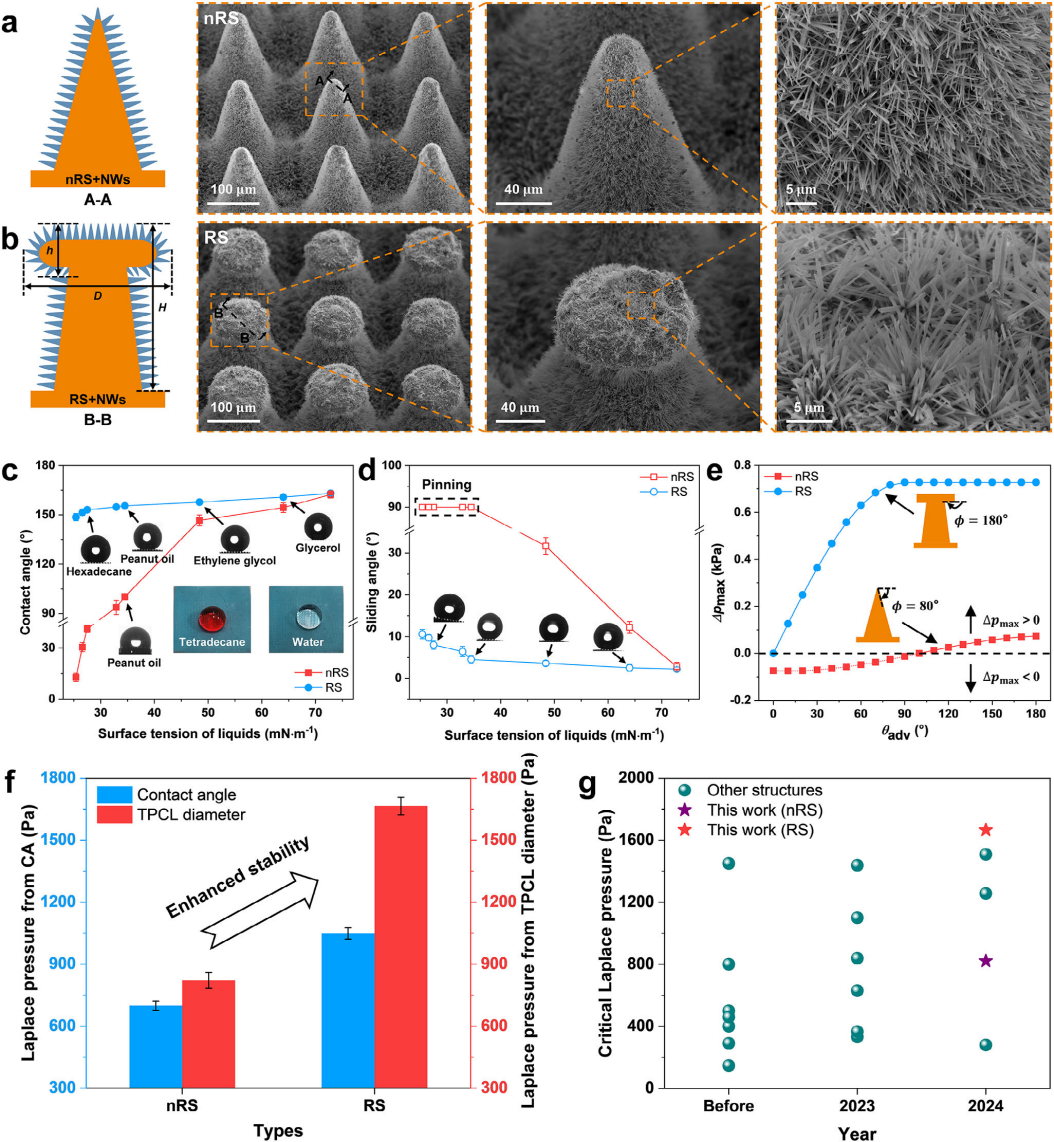
RSs designed and fabricated in this study for anti‐icing, their fundamental liquid‐repellent properties, and the room‐temperature CB stability. Schematic and SEM images at different magnifications: a) nRS; b) RS. c,d) Contact angles and sliding angles of liquids with different surface tensions on nRS and RS surfaces. Insets show photographs of a tetradecane (red) droplet and a deionized water (transparent) droplet on RS surface. e) The relationship between breakthrough pressure and apparent advancing contact angle θ_*adv*_ for both surfaces. f) Critical Laplace pressures derived from contact angles and TPCL diameters for both surfaces at room temperature: nRS and RS surfaces. g) Comparison of the critical Laplace pressure between our fabricated surfaces and other reported surfaces. Detailed data are presented in Figure S9 (Supporting Information). Data in (c,d,f) are mean ± s.d. from at least three independent measurements.

We developed a novel manufacturing method combining laser, hot‐pressing, and chemical treatment, which is detailed in the Experimental Section and Table S1 (Supporting Information). Periodic RS with hierarchical scales was fabricated on a copper plate surface. The structure consists of micrometer‐scale T‐shaped pillars densely covered with NWs (RS+NWs will be abbreviated as RS in the following text) (Figure 2b; Figure S1, Supporting Information), effectively achieving synergistic enhancement of both the nano‐effect and the re‐entrant mechanism. Specifically, the micro T‐shaped arrays endow the surface with superomniphobic properties and a stabilized CB state through their unique overhanging features, while the surface nanostructures serve as a secondary reinforcement factor that further enhances these characteristics. Additionally, we prepared a control surface composed of a microcone array with dense NWs, representing a traditional non‐re‐entrant structure (nRS, nRS+NWs will be abbreviated as nRS), as depicted in **Figure** **2**a. It can be observed that the tightly and uniformly distributed NWs can reach lengths of 15–20 µm, with diameters of ≈50–300 nm. At the microscale, the heights of the two surface microstructures are consistent, both being 200 µm. The cap diameter (*D*) of RS is ≈85 µm, and the pillar spacing (*P*) is 180 µm. After modification with fluoroalkylsilane, EDS elemental analysis revealed a significant increase in the surface F element content (Figure S2, Supporting Information). At this stage, both surfaces exhibited excellent superhydrophobic properties. The contact angles of RS and nRS surfaces were 163.1 ± 1.4° and 162.4 ± 1°, respectively, while the sliding angles were 2.2 ± 0.2° and 2.8 ± 0.4°, respectively (Figure S3, Supporting Information). We also tested the repellency of nRS and RS surfaces to various liquids. Figure 2c,d show the variations in contact angles and sliding angles as functions of liquid surface tension for both surfaces. It was found that multiple liquids, including tetradecane, could maintain the CB state on RS surface, with apparent contact angles greater than 150° and sliding angles less than 10° (Figure S4, Supporting Information). In contrast, nRS surface fails to repel low‐surface‐tension liquids, which undergo immediate pinning upon contact. These results demonstrate that RS surface exhibits strong liquid repellency.

However, by continuously increasing the hydrostatic pressure within the liquid phase, the liquid front will inevitably break through the structure and continue to penetrate. According to the condition where the pressure difference across the meniscus is balanced by the surface tension at the contact line (detailed analysis is provided in Supporting Discussion 1.1), the breakthrough pressure of the wetting transition induced by the two structures can be expressed as^[^ ^37^ ^]^:

(1) $$\Delta p_{\max}=\gamma \frac{2\pi h_{1}\tan\frac{\alpha }{2}}{p^{2}-\pi h_{1}^{2}\tan^{2}\frac{\alpha }{2}}\sin\left(\theta_{\mathrm{adv}}-\frac{\pi }{2}-\frac{\alpha }{2}\right)\mathrm{nRS}$$

(2)

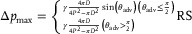

where *γ* represents the surface tension of the liquid; *h*_1_ and *α* denote the descent height of the liquid‐air‐solid triple‐phase contact line (TPCL) and the taper angle of nRS, respectively; *D* and *P* are the cap diameter and pillar spacing of RS; and θ_adv_ represents the apparent advancing contact angle of the liquid on the sidewalls of the microstructure (Figure S5, Supporting Information). Based on Equations (1) and (2), we calculated the breakthrough pressures for both surfaces at various θ_adv_ values, as plotted in Figure 2e. For deionized water, RS surfaces exhibit a breakthrough pressure over 19 times higher than that of nRS surfaces. The results demonstrate that RS surfaces consistently provide positive breakthrough pressures (> 0) regardless of liquid wettability, reaching the geometrically allowed maximum when $\theta_{\mathrm{adv}}\geq \;\frac{\pi }{2}$. In contrast, nRS surfaces exhibit a transition from negative to positive breakthrough pressures with increasing θ_adv_. This indicates that nRS can only sustain the CB state for liquids forming high contact angles with the substrate material.

Here, we controlled the pillar height (*H*), pillar spacing (*P*), and cap diameter (*D*) as variable structural parameters. The effects of different microstructural dimensions of RS surface on contact angle and ice adhesion strength were investigated (Figure S6, Supporting Information). The results indicate that larger *D* and smaller *P* contribute to a longer TPCL, consequently reducing the contact angle while increasing ice adhesion strength. However, according to Equation (2), an increase in *D* and a decrease in *P* result in a higher breakthrough pressure. To balance the conflicting structural demands, we selected 85 and 180 µm as the optimal dimensions for the cap diameter and pillar spacing, respectively. Additionally, RS surfaces with different pillar heights were prepared. Considering the trade‐off between the stability of the CB state and the mechanical robustness of the structure, the height of the micro T‐shaped array was determined to be 200 µm.

The size of droplets can influence their wetting state on superhydrophobic surfaces. Using the structurally optimized RS surface, we investigated the pressure resistance of shrinking evaporating droplets in maintaining the CB state on both surfaces (Δ*p*  = 2γ/*R* , where Δ*p* represents the Laplace pressure, *γ* is the liquid surface tension, and *R* is the droplet radius). Figure S7 (Supporting Information) shows the gradual evaporation process of droplets on the control surface and RS surface over time. Initially, evaporation exhibited similar behavior on both surfaces. The TPCL of the droplets remained pinned, while the contact angle continuously decreased, indicating a constant contact radius (CCR) evaporation mode. As evaporation progressed, the TPCL depinned and retracted to the next microstructure, causing a sudden decrease in the contact diameter and a temporary increase in the contact angle. Subsequently, the evaporation mode on RS surface transitioned to a mixed mode with simultaneous changes in TPCL diameter and contact angle. In contrast, nRS surface primarily followed the CCR mode, with both surfaces undergoing continuous pinning and depinning of the contact line. The critical Laplace pressure of RS surface when the TPCL diameter tended to stabilize was 1665.4 ± 42 Pa, significantly higher than that of nRS surface at 820.6 ± 38 Pa (Figure 2f; Figure S8, Supporting Information). We compared the critical Laplace pressures of both surfaces with those reported in some studies providing data (Figure 2g). It can be observed that RS can better enhance the stability of the CB state at room temperature. In the following sections, we will systematically evaluate their anti‐icing performances, including delay icing, ice adhesion strength, and icing and melting cycles.

### 2.2 Prolonged Icing Delay and Anti‐Frosting Performances of the RSs

**Figure 3:**
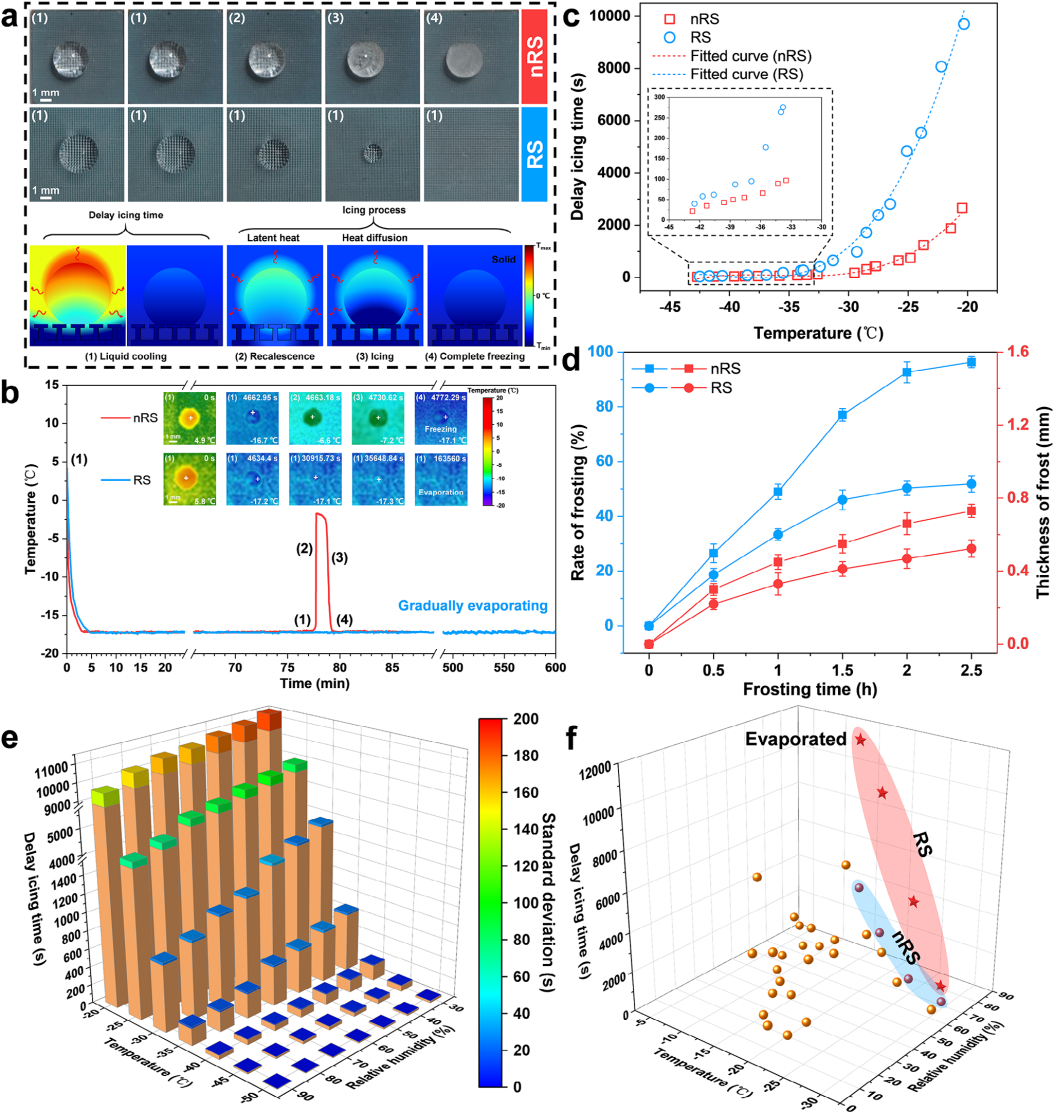
Prolonged icing delay and anti‐frosting performances of the RSs are fabricated. a) Optical images of different icing stages of droplets on nRS and RS surfaces, and thermographic schematics showing the temperature distribution of the icing process on RS surface from the side‐view. The ambient temperature was ‐17 ± 2 °C, and the humidity was controlled at 70 ± 5% RH. b) Relationship between droplet temperature and time on both surfaces. Insets show top‐view thermal images of droplets at different icing stages on both surfaces. The icing conditions are the same as in (a). c) Delay the icing time of droplets on both surfaces at different temperatures. Data between −45 °C and −33 °C are shown in the enlarged box. Humidity was controlled at 70 ± 5% RH. d) The effect of static frosting time on the frosting rate and the thickness of the frost. e) Delay icing time of RS surface under different temperature and humidity conditions. f) Comparison of delay icing time between our two surfaces and other reported surfaces under different environmental conditions. Data for RS and nRS surfaces are highlighted in red and blue regions, respectively, while data for other surfaces are marked with golden spheres. Detailed data are presented in Figure S15 (Supporting Information).

To investigate the low‐temperature CB stability and anti‐icing performance of the two surfaces, we conducted icing delay experiments under various environmental conditions. Here, we designed a low‐temperature freezer that allows convenient observation and recording of the icing behavior of droplets under different temperatures and humidities (Figure S10, Supporting Information). The icing process of a droplet can generally be divided into four stages: 1) droplet cooling; 2) recalescence; 3) icing process; 4) complete freezing.^[^ ^14^ ^]^ **Figure** **3**a shows optical snapshots of droplets at different icing stages on the two surfaces. Droplets on nRS surface successively underwent the four icing stages and rapidly froze. In contrast, the droplet on RS surface remained in stage (1) and gradually evaporated until it disappeared. We also provided thermographic schematics of the droplet icing process on RS surface (Figure 3a), defining the duration from the start of cooling to the occurrence of recalescence as the delay icing time. Figure 3b shows the variation of droplet temperature over time on both surfaces. It was observed that the delay icing time on nRS surface was 4662.9 s at −17 °C. In contrast, RS surface consistently maintained droplets in a stable supercooled state for over 600 min until complete evaporation in at least three repeated experiments. Moreover, the captured thermographic top views of droplets on the two surfaces exhibited the same icing stages as discussed earlier. These results strongly validate the effectiveness of RS in enhancing CB state stability at low temperatures.

We compared the delay icing time of the two surfaces at different temperatures to illustrate the enhanced anti‐icing performance of RS surface. As shown in Figure 3c, both surfaces inevitably experienced rapid icing at temperatures below −33 °C. However, compared to nRS surface, RS surface consistently exhibited superior delay icing performance. As the temperature increased, the difference in delay icing time between the two surfaces became more pronounced. To elucidate the synergistic effects between the nanostructures and the microscale re‐entrant features, we also directly prepared NWs on flat copper surfaces. By comparing the icing delay performances of the RS versus the NW surfaces at different low temperatures, it was shown that the RS surface demonstrated significantly prolonged icing delay compared to the flat surface with the NWs (Figure S11, Supporting Information). This result conclusively verifies the critical role of the microscale re‐entrant features in enhancing anti‐icing performance. In addition to the delay icing time of droplets, condensation frosting is another challenge in practical anti‐icing applications.^[^ ^38^ ^]^ We controlled the surface temperature to decrease from room temperature to −15 °C, with humidity maintained at 80 ± 5% RH, and recorded the frosting on two sample surfaces from top and side views every 0.5 h (Figure 3d; Figure S12, Supporting Information). The rate of frosting of nRS surface increased to 49% within 1 h and even reached 96% after 2.5 h, with the entire surface gradually frosting from the outside to the inside, almost completely covered by frost. In contrast, the rate of frosting for RS surface was 52% after 2.5 h, with an obvious frost‐free area remaining in the central region. In addition, the frost thickness on nRS surface was consistently greater than that on RS surface, reaching 1.4 times that of RS surface after 2.5 h. These observations indicate that RS surface exhibits excellent static anti‐frosting performance.

To further explore the threshold for the anti‐icing performance of RS surface, we tested the delay icing time of RS surface under different temperatures and humidities (Figure 3e). It was found that the delay icing time decreased rapidly below −35 °C, indicating that extremely low temperatures disrupt the CB state stability of RS surface. Although increased humidity also negatively affects delay icing, its impact on the anti‐icing performance of RS surface is less significant compared to temperature. If we set the delay icing time for 300 s as the benchmark of determining anti‐icing capability, we can establish the limiting conditions for anti‐icing performance based on temperature and humidity (Figure S13, Supporting Information). When the temperature is above −30 °C, RS surface can maintain superior delay icing performance regardless of humidity. At humidity levels below 60% RH, the temperature requirement for maintaining anti‐icing performance can be slightly relaxed. These findings can better guide the practical application of RS surface.

To elucidate the intrinsic mechanism behind the variation in anti‐icing performance between the surfaces, we established a computational model for the air pocket pressure of nRS and RS by analyzing the thermodynamic changes at the liquid‐air interface based on the ideal gas law (detailed derivation process is provided in Supporting Discussion 1.2). Based on the established model, we obtained the relationship between air pocket pressure and temperature for both surfaces (Figure S14, Supporting Information). The air pocket pressure increases with decreasing temperature on both surfaces, and RS surface is consistently higher than that of nRS surface. At a temperature of 15 °C, the air pocket pressure of RS (31.8 kPa) is more than three times that of nRS (10.4 kPa), and the difference between them gradually increases as the temperature decreases. The air pocket pressure contributes to the low‐temperature CB state stability of the surfaces. A higher air pocket pressure can better prevent the descent of the liquid‐air interface, thereby slowing down the increase in solid‐liquid contact area and the reduction of interfacial thermal resistance. This significantly increases the delay icing time and ensures better anti‐icing performance of RS surface. As shown in Figure 3f, we compared the two surfaces with other anti‐icing surfaces reported in the literature. It was observed that RS surface significantly prolongs the icing time under various conditions and outperforms the compared surfaces. Particularly under environmental conditions of −17 °C and 70% RH, RS surface can delay icing time until the droplet completely evaporates. The theoretical and experimental results above provide substantial evidence that RS can enhance anti‐icing performance.

### 2.3 Enhanced Icephobicity and Anti‐Ice‐Pinning Performances of the RSs

**Figure 4:**
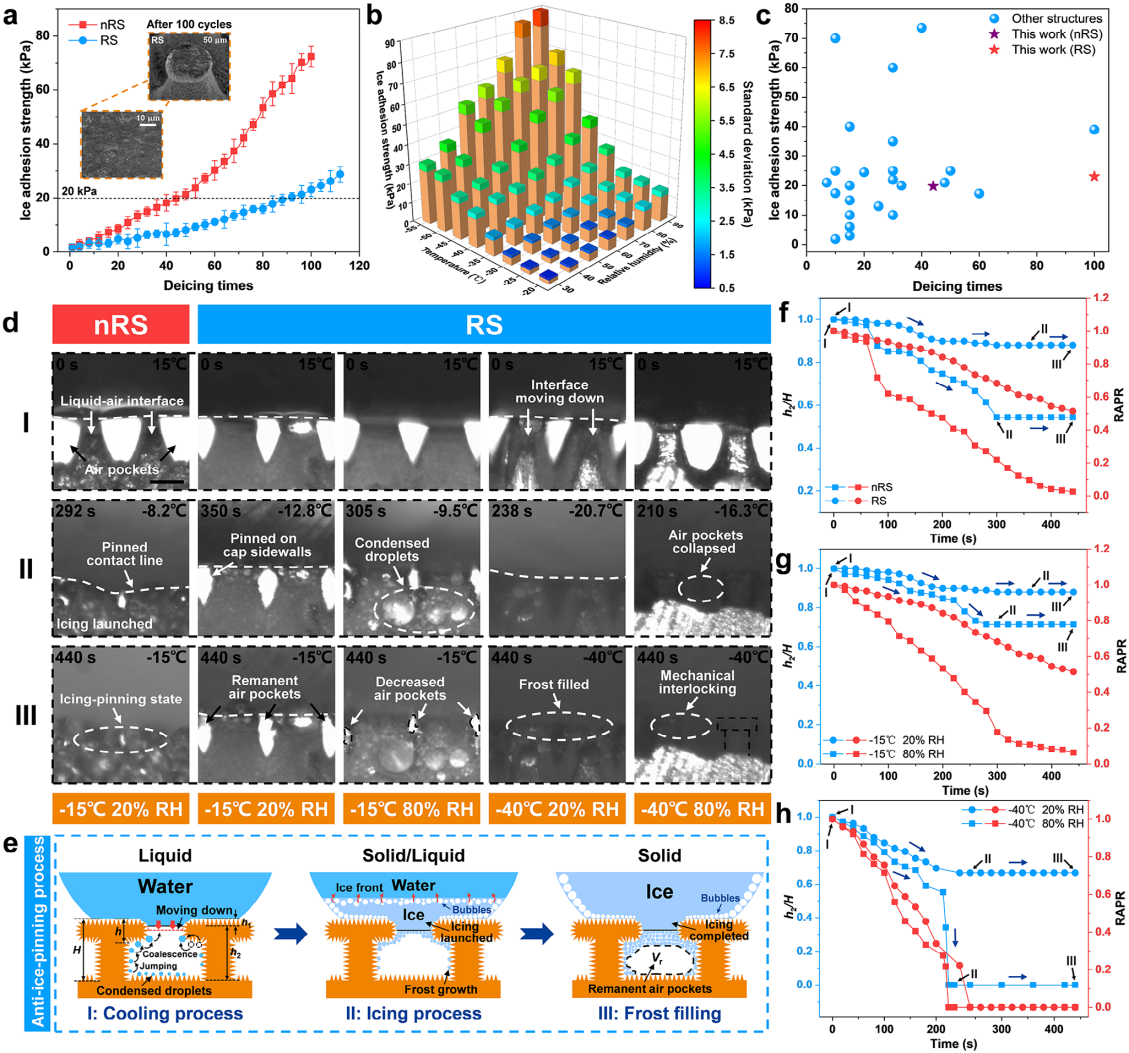
Enhanced icephobicity and anti‐ice‐pinning performances of the RSs were fabricated. a) Effect of icing and deicing cycles on the ice adhesion strength of RS and nRS surfaces. The inset shows an SEM image of a single micro‐nanostructure on RS surface after 100 deicing cycles. b) Ice adhesion strength of RS surface under different temperatures and humidities. c) Comparison of ice adhesion strength after multiple deicing cycles between our fabricated surfaces and other reported surfaces. Detailed data are presented in Figure S21 (Supporting Information). d) Side‐view observation images of the changes in the triple‐phase interface and air pockets on both surfaces under different environmental conditions. The scale bar is 100 µm. e) Schematic illustration of the anti‐ice‐pinning process on RS surface. f) Changes in the pinning remaining height ratio (*h*_2_/*H*) and remaining air pocket volume ratio (RAPR) over icing time for both surfaces. The surface temperature was decreased from 15 °C to −15 °C, with the ambient temperature and humidity controlled at 15 ± 2 °C and 20 ± 5% RH, respectively. g,h) Changes in *h*_2_/*H* and RAPR over icing time for RS surface under four environmental conditions. The temperature and humidity conditions correspond to those in (d).

Another essential property of an ideal superhydrophobic anti‐icing surface is easy deicing. The ice adhesion strength after icing can be used to evaluate this property. We conducted multiple icing‐deicing cycle experiments (**Figure** **4**a). The results show that nRS and RS surfaces exhibited similar initial ice adhesion strengths. However, as the number of deicing cycles increased, the ice adhesion strength of nRS surface obviously increased, reaching 72.4 ± 3.9 kPa after 100 deicing cycles. In contrast, the ice adhesion strength of RS surface increased slowly, remaining below 25 kPa even after 100 deicing cycles. This demonstrates the excellent icephobic durability of RS surface. To further analyze the impact of surface damages on the icephobic durability, we characterized the microstructural morphologies of both the nRS and RS surfaces after 100 deicing cycles (Figures S16 and S17, Supporting Information). The observations reveal the complete disappearance of NWs on the upper nRS surface, also with wear damages at the tops of some microcones. This hierarchical degradation of micro‐nanostructures directly accounts for the significant deterioration in the icephobicity of the nRS surfaces. In contrast, repeated deicing only removed large‐scale NWs from the RS tops, while microscopic roughness features on the cap surface (rather than NWs) remained preserved. Crucially, the micrometer‐scale T‐shaped framework retained integrity, with undamaged abundant nanostructures on both its sidewalls and bases. These preserved multiscale features enable effective liquid‐penetration inhibition during icing, thereby rendering the RS surfaces prolonged low ice adhesion strength. Compared to the EDS images of the pristine state, we noted a decrease in the content of low‐surface‐energy elements F and Si on the surface, which aligns with the trend of increasing ice adhesion strength after multiple deicing cycles. However, the content and distribution of other elements remained largely unchanged (Figure S18, Supporting Information). The structural and chemical robustness ensures the reusable icephobicity of RS surface.

We also investigated the threshold of the deicing performance for RS surface. Figure 4b shows the ice adhesion strength of RS surface under different temperatures and humidities. The results indicate that the ice adhesion strength increases with decreasing temperature and increasing humidity. However, the ice adhesion strength is more significantly affected by temperature. It has been reported that ice can be removed from the surface under slight disturbances such as wind or vibration when the ice adhesion strength is below 20 kPa.^[^ ^39^ ^]^ Using 20 kPa as the benchmark for easy deicing, we found that when the temperature is below −30 °C, the humidity range needs to be appropriately limited to ensure the deicing performance of RS surface. However, when the temperature is above −30 °C, humidity does not affect the ice adhesion strength of the surface (Figure S19, Supporting Information). A collective comparison of the hydrophobicity and icephobicity of different surfaces demonstrates that the surfaces we fabricated exhibit excellent deicing capabilities (Figure S20, Supporting Information). Moreover, to evaluate the superiority of icephobic durability, we compared the ice adhesion strength after multiple deicing cycles of our fabricated surfaces with those reported in other studies, as shown in Figure 4c. It is evident that RS surface maintains a low ice adhesion strength even after the maximum number of icing and deicing cycles, indicating that RS possesses exceptional icephobic durability.

To reveal the kinetic mechanisms of anti‐icing performances influenced by surface structures and environmental conditions, as well as the failure conditions of anti‐ice‐pinning, we observed the dynamic evolution of the triple‐phase interface and air pockets on both surfaces under different environmental conditions, as shown in Figure 4d, Figure S22, and Videos S1 and S2 (Supporting Information). The surface temperature of the samples was decreased from 15 °C to −15 °C or −40 °C and then maintained. The ambient temperature and humidity were controlled at 15 ± 2 °C and the humidity indicated in the figure, respectively. When the temperature was reduced to −15 °C and the humidity was maintained at 20% RH, droplets on nRS surface gradually penetrated the micro‐nanostructures during the cooling process. The liquid‐air interface stopped descending before icing occurred. After complete freezing, frost filled the gaps and formed ice‐pinning. In contrast, on RS surface, the liquid‐air‐solid triple‐phase interface was arrested at the sidewalls of the caps during continuous downward movement until icing launched. Significant air pockets remained within the gaps of the microstructures after complete icing. For RS surface, when the humidity increased, numerous condensed droplets appeared on the outer surfaces of the microstructures. Adjacent tiny droplets coalesced and condensed into frost during cooling, leading to a continuous reduction in the air pocket volume. Eventually, the increased frost completely filled the gaps. When the temperature was reduced to −40 °C, RS surface lost the air pockets within the structural gaps after complete icing, regardless of humidity. The further descent of the triple‐phase interface and the sudden increase in frost at low temperatures caused the loss of air pockets at lower humidity levels. At higher humidity levels, the synergistic effect of low temperature and high humidity promotes droplet breakthrough and subsequent collapse.

Additionally, we summarized the anti‐ice‐pinning process on RS surface (temperature and humidity were −15 °C and 20% RH, respectively) (Figure 4e). It can be divided into three stages: I) Cooling process; As the temperature decreased, the liquid‐air interface moved downward. Subsequently, condensation droplets appeared and underwent coalescence and jumping.^[^ ^40^ ^]^ When the liquid‐air interface approached the lower corners of the cap sidewalls, TPCL was arrested until icing launched. II) Icing stage; Once the temperature dropped below the freezing point, droplet freezing occurred instantaneously. The triple‐phase interface remained stationary during the icing process. Condensate within the microstructure gaps began to frost and grow. III) Frost filling; From the onset of condensation frosting to the end of the cooling and constant temperature stage, frost continuously filled the gaps of the microstructures, causing the air pocket volume to decrease. Notably, the effective pinning of the liquid droplet at the lower corner of RS cap during the icing process is the primary factor hindering the continuous downward movement of the triple‐phase interface. This localized pinning generates additional structural drag force and a higher energy barrier, thereby preventing further liquid penetration and ensuring higher CB state stability and better anti‐ice‐pinning performance.

We defined the ratio of the remaining air pocket volume to the initial air pocket volume as the remaining air pocket volume ratio (RAPR). Simultaneously, the depth of liquid penetration and the remaining height of the structure were defined as *h*_1_ and *h*_2_, respectively. To better compare the ice‐pinning resistance of different structures under various environmental conditions, we measured the changes in $\frac{h_{2}}{H}$ and RAPR over time during the entire icing process. It was found that the $\frac{h_{2}}{H}$ and RAPR of RS surface were consistently greater than those of nRS surface (Figure 4f; Figure S23, Supporting Information). The depth of liquid penetration determines the contact area between the microstructures and ice, thereby influencing the ice adhesion strength. This explains why the ice adhesion strength of RS surface is lower than that of nRS surface under the same conditions. Figure 4g and Figure S24 (Supporting Information) show that at a temperature of −15 °C, *h*_2_ under 20% RH was greater than that under 80% RH, and the difference in remaining air pocket volume between the two conditions was even more significant. This is attributed to the increased condensation droplets and frost at high humidity. The coalescence and jumping of more condensed droplets affected the pinning depth. Meanwhile, increased frost severely reduced the gas amount within the microstructure gaps. The results in Figure 4h; Figure S24 (Supporting Information) indicate that when the temperature was reduced to −40 °C and the humidity was maintained at 20% RH, the structure still retained a relatively large *h*_2_, but the gaps were completely filled with frost generated at low temperatures. Furthermore, under the combined effect of −40 °C and 80% RH, the air pockets on RS surface suddenly collapsed during the cooling process, leading to a sharp decrease in *h*_2_ and RAPR. The remaining air pocket volume indicates whether the droplets remain in the CB state. Therefore, these experimental results also confirm that RS surface possesses enhanced low‐temperature CB stability.

We utilized the energy changes during the wetting processes of droplets on different surfaces to explain the hindering effect of the special re‐entrant feature of RS surface on liquid penetration. The wetting processes of droplets on the two surfaces exhibit significant differences (Figure S25, Supporting Information). Compared to nRS surface, the curvature variation of RS surface in the vertical direction makes the droplet wetting process more complex. We developed theoretical equations to describe the nondimensionalized free energy changes during the droplet penetration processes on the two surfaces. The detailed derivation of the model is provided in Supporting Discussion 1.3. Droplets on RS surface tend to be arrested at the lower corners of the microstructured caps. This increases the energy barrier hindering the transition from the CB state to the Wenzel state. Droplets need to overcome an additional cap barrier to continue the wetting process. We provided the formulas for calculating the nondimensionalized total energy barrier and the energy barrier when the penetration depth equals the cap thickness for the wetting transition on nRS and RS surfaces. Figure S26 (Supporting Information) shows a comparison of the nondimensionalized total energy barriers and the energy barriers at the caps for both surfaces. We found that the total energy barrier for the wetting process on RS surface is ≈2.7 times that of nRS surface. Additionally, when TPCL moves the cap thickness distance along the sidewalls of the structures on both surfaces, the energy barrier of RS surface is 3.2 times that of nRS surface. This undoubtedly demonstrates the critical role of the re‐entrant feature in increasing the energy barrier during the wetting transition process, and the densely distributed nanostructures on the microstructures further enhance this effect.^[^ ^8^ ^]^ With the energy barrier significantly increased, the transition of droplets on RS surface from the CB state to the Wenzel state becomes more difficult, thereby ensuring the low‐temperature CB stability and anti‐ice‐pinning performance of the surface.

### 2.4 Ice‐Melting‐Triggered Spontaneous Dewetting in the RSs

**Figure 5:**
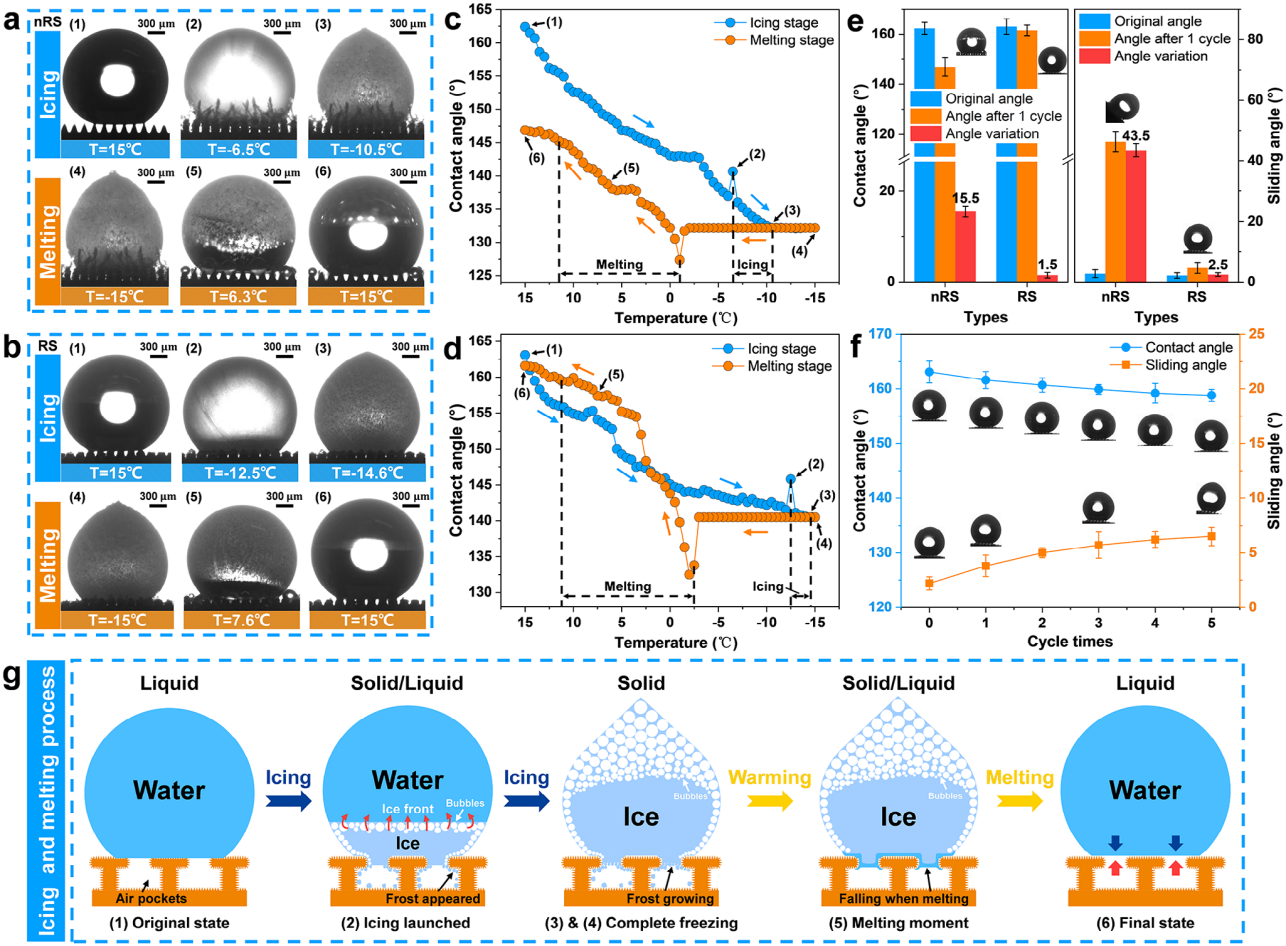
Reversible icing/melting behaviors and spontaneous dewetting capability of the RSs are fabricated. Side‐view experimental observations of droplet states and the relationship between contact angle and temperature during the icing‐melting process: a,c) nRS surface.(b,d) RS surface. Icing and melting stages are divided by black dashed lines. Blue and orange arrows indicate the circulation directions of icing and melting processes, respectively. The fluctuations of the correlation evaluations are described in the Experimental Section. e) Changes in the contact angle and sliding angle of droplets on both surfaces after icing‐melting cycles. f) The effect of the number of icing and melting cycles on the contact angle and sliding angle of droplets on RS surface. The insets show the contact and sliding states of droplets after different numbers of icing and melting cycles. g) Schematic illustration of icing and melting processes on RS surface.

We employed icing and melting cycle experiments to evaluate the spontaneous dewetting capability on RS surface after ice melting. **Figures** **5**a–d and Figure S27 (Supporting Information) illustrate the droplet states, contact angles, and contact diameters of the two surfaces as functions of temperature during icing and melting processes. It can be observed that during the icing process, the CB state on both surfaces is gradually disrupted, leading to a continuous decrease in contact angles and an increase in contact diameters. However, the rate of contact angle decrease and contact diameter increase on RS surface is lower than that on nRS surface. Consequently, when cooled to the same temperature, the contact angle of droplets on RS surface remains consistently larger than that on nRS surface, while the contact diameter remains consistently smaller (Figure S28, Supporting Information). As the temperature rises, the ice droplets on both surfaces show no significant changes until melting occurs. Subsequently, both surfaces exhibit the same trend of increasing contact angles and decreasing contact diameters. It should be noted that, compared to nRS surface, droplets on RS surface almost completely recover to the initial state after the icing and melting cycle. The detailed process is shown in Videos S3 and S4 (Supporting Information).

After one icing‐melting cycle, the contact angle of droplets on nRS surface decreased by 15.5° ± 1.2°, while the sliding angle increased by 43.5° ± 2.2°, indicating that the air pocket loss induced by icing on nRS surface cannot be restored after melting. In contrast, a sliding angle of only 4.7° after the icing and melting cycle ensures that droplets on RS surface can rapidly detach (Figure 5e). To characterize the degree of CB state recovery after the icing and melting cycle, we compared the contact angle retention degree (CARD) and contact diameter retention degree (CDRD) of droplets on different surfaces (see S29 for detailed definitions). Clearly, the CARD and CDRD of droplets on RS surface can reach 99.1% and 98.3%, respectively, significantly higher than those on nRS surface. These results confirm that droplets on RS surface can spontaneously recover to the CB state with a high recovery rate after icing and melting cycle.

To fully understand the mechanism of spontaneous CB state recovery after the icing‐melting cycle, we summarized the schematic diagram of icing and melting processes of the droplet on RS surface, as shown in Figure 5g. Initially, the droplet on RS surface is in the CB state, with air pockets trapped within the structural gaps. As the temperature decreases, the air pockets and ambient air gradually dissolve into the droplet, and the liquid begins to penetrate downward. When icing occurs, the bottom of the droplet in contact with the microstructures instantaneously solidifies, while the top remains liquid. Under the influence of volume contraction at the bottom, the contact angle suddenly increases, and the contact diameter suddenly decreases. Meanwhile, the icing process is accompanied by continuous frosting. After complete icing, the droplet on RS surface cannot maintain the CB state due to the loss of air pockets caused by the downward shift of the triple‐phase interface and frost filling. When the temperature rises to the melting point, the bottom of the ice droplet melts first, forming liquid water. At the same time, the frozen bubbles continuously and rapidly move along the edge of the ice droplet to the melting front and eventually escape into the micro‐nanostructures, promoting the formation and recovery of air pockets. Ultimately, the droplet transitions back to the CB state after complete melting.

To demonstrate the robustness of the spontaneous CB state transition of droplets on RS surface, we conducted multiple icing and melting cycle experiments. As the number of cycles increases, the contact angle of droplets on RS surface gradually decreases, and the sliding angle gradually increases. However, after 5 icing‐melting cycles, the contact angle and sliding angle of the droplets can still be maintained at 158.8° ± 1.1° and 6.5° ± 0.9° (Figure 5f). At this point, the CARD and CDRD of the droplets reach 97.4% and 93.7% (Figure S29, Supporting Information). This indicates the robustness of the spontaneous transition from the Wenzel state to the CB state of droplets on RS surface after icing and melting cycles.

### 2.5 Overall Anti‐Icing Regimes of the RSs

**Figure 6:**
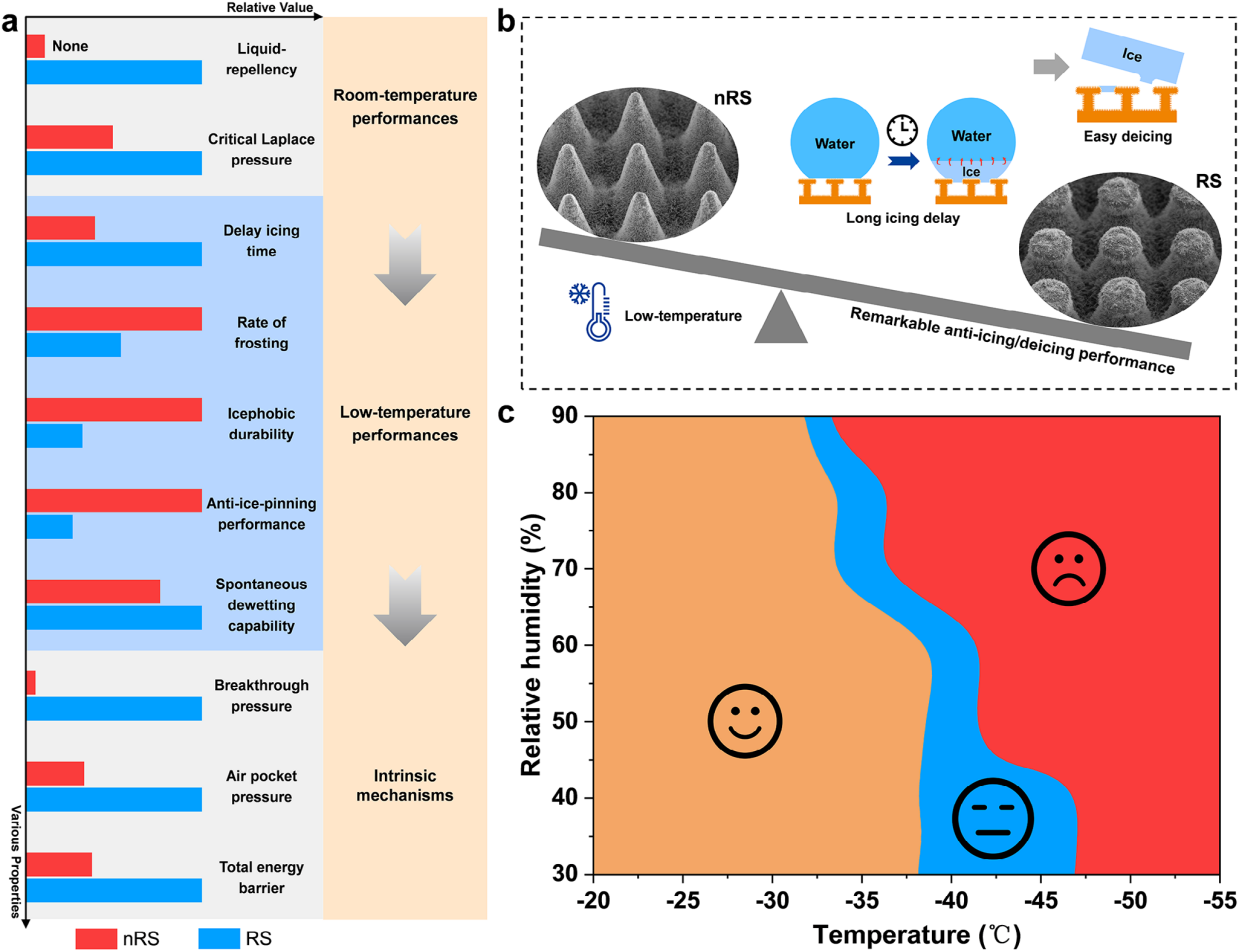
A sketchout of the anti‐icing performances of the RSs and the essential regimes to keep them effective. a) Comparison of room‐temperature performances, low‐temperature behaviors, and underlying mechanisms between nRs and RS. b) Visualized comparison of the anti‐icing/deicing performance between nRS and RS. c) Critical freezing condition window for effective anti‐icing performances of the RSs. Smiling: anti‐icing; Neutral: partial anti‐icing; Frowning: anti‐icing failure.

To comprehensively compare the performance of the fabricated nRS and RS surfaces, we selected various properties ranging from room temperature to low temperature for integrated evaluation, including liquid‐repellency, critical Laplace pressure, delay icing time, static anti‐frosting rate, icephobic durability, percentage of pinning depth, and CARD, as shown in **Figures** **6**a and Figure S30 (Supporting Information). At room temperature, RS surfaces exhibit superior liquid repellency absent in nRS surfaces, capable of repelling various organic liquids, including tetradecane. Evaporation experiments further demonstrate that RS surfaces possess significantly higher critical Laplace pressure than nRS surfaces, indicating enhanced room‐temperature CB state stability. Moreover, RS surfaces exhibit an ultra‐long delay icing time, ideal anti‐frosting performance, low ice adhesion strength after multiple deicing cycles, exceptional anti‐ice‐pinning properties, and outstanding ice‐melting‐triggered spontaneous dewetting capability. To uncover the intrinsic mechanism behind the remarkable anti‐icing performances of RS surfaces, we established a series of theoretical models. The results reveal that RS surfaces possess higher room‐temperature breakthrough pressure, improved low‐temperature air pocket pressure, and greater nondimensionalized total energy barriers during wetting transitions (Figure 6a). Based on the comprehensive performance evaluation summarized above, we present a visualized comparison of the anti‐icing/deicing performance between nRS and RS (Figure 6b). In low‐temperature environments, RS surfaces significantly delay icing time while maintaining ultra‐low ice adhesion strength after icing. We therefore conclusively reveal the remarkable anti‐icing/deicing performance of RS surfaces. These findings demonstrate the outstanding comprehensive performance and substantial practical application potential of RS surfaces.

In addition, by combining the anti‐icing/deicing performance thresholds from Figures S13 and S19 (Supporting Information), we determined the critical freezing condition window for effective anti‐icing performances of RS surfaces (Figures 6c; Figure S31, Supporting Information). RS surfaces maintain robust CB state stability and superior anti‐icing performances above −30 °C, attributable to enhanced air pocket stabilization. When the temperature drops below −50 °C, the extreme cold triggers a catastrophic collapse of air pocket stability, resulting in complete anti‐icing failure. In the temperature range between −30 °C and −50 °C, the anti‐icing performances become partially compromised due to the synergistic effects of temperature and humidity, and the exact failure mechanisms require further investigation. Collectively, these insights provide a scientific framework for deploying RS surfaces across diverse climatic conditions.

## 3 Conclusion and Outlook

In this study, a comprehensive assessment on the anti‐icing performances of RSs has demonstrated their robust CB state stability and excellent anti‐icing properties at low temperatures, while revealing the underlying mechanism for performance enhancement. Through an engineering approach, we designed and fabricated RSs directly on metal surfaces with precise control over dimensional parameters. The manufactured RS surface not only exhibits an outstanding critical Laplace pressure of 1665.4 ± 42 Pa but also demonstrates remarkable anti‐icing performances, with a delay icing time exceeding 600 min at −17 °C and an ice adhesion strength of only 1.6 ± 1.4 kPa. Remarkably, the surfaces maintain ice adhesion strength below 25 kPa after 100 cycles of icing and deicing, indicating their exceptional icephobic durability. In situ observations of triple‐phase interface movement during the icing process revealed that droplets preferentially pin at the lower corners of microstructure overhangs, thereby synergistically increasing both thermodynamic energy barriers and structural drag force to effectively prevent CB‐to‐Wenzel transition. Furthermore, icing‐melting cycle experiments confirmed the spontaneous dewetting transition from Wenzel to CB state upon temperature increase. Overall, this work establishes a comprehensive framework for understanding, developing, and engineering robust super‐repellent surface micro/nanostructures for anti‐icing. The methodology, while demonstrated on copper, can be broadly applicable to other metals and even non‐metal materials, providing a general strategy and further opportunities for advancing functional micro/nanostructures toward practical applications.

## 4 Experimental Section

### Materials

Commercially available copper plates (purity ≥ 99.9%) were used as the substrate for processing. 1H,1H,2H,2H‐perfluorodecyltrimethoxysilane (PFDTS) was purchased from Shanghai Meryer Biochemical Technology Co., Ltd, China. Sodium hydroxide and ammonium persulfate, which needed to be prepared as solutions, were obtained from Shanghai Titan Scientific Co., Ltd, China. Deionized water (*γ*  = 72.8 mN m^−1^), glycerol (*γ* = 64 mN m^−1^), ethylene glycol (*γ* = 48.4 mN m^−1^), hexadecane (*γ* = 27.5 mN m^−1^), tetradecane (*γ* = 26.6 mN m^−1^), and dodecane (*γ* = 25.4 mN m^−1^) were all purchased from Shanghai Macklin Biochemical Technology Co., Ltd, China. Paraffin oil (*γ* = 32.9 mN m^−1^) and peanut oil (*γ* = 34.5 mN m^−1^) were purchased from Shanghai Aladdin Biochemical Technology Co., Ltd, China, and Luhua Group, China, respectively. The superomniphobic properties of the sample surface were tested using the above‐mentioned liquids.

### Micro‐Nanostructure Fabrication Method

First, copper plates were cut into dimensions of 20 mm × 20 mm × 1 mm. The samples were then polished to a mirror finish and ultrasonically cleaned in ethanol for 5 min. A YDFLP‐200 nanosecond laser system, with a pulse width of 150 ns, a central wavelength of 1064 nm, and a repetition rate of 300 kHz, was used to fabricate the microstructures on the sample surface. In an atmospheric environment, the focused laser beam was scanned across the sample surface in a cross‐line pattern using an x‐y galvanometer. The spacing between the laser scan paths was 10 µm, and the scanning speed was 2000–3000 mm s^−1^. The center‐to‐center spacing of the fabricated microcone arrays was 180 µm, with the diameter of the unscanned regions being 60 µm. After ablation, the samples were ultrasonically washed in ethanol and deionized water, respectively. Based on the fabricated microcone arrays, the samples were hot‐pressed to obtain RSs. The heating temperature and pressure varied depending on the number and dimensions of the microcone arrays. When the hot‐pressed samples cooled to room temperature, they were sequentially ultrasonically treated in dilute sulfuric acid and ethanol for 5 min each to remove oxides from the microstructure surfaces and to clean the samples. Subsequently, the sample was dried using high‐pressure nitrogen gas. To generate abundant nanostructures, the samples were immersed in a 100 mL mixed solution of 2.5 mol L^−1^ NaOH and 0.1 mol L^−1^ (NH_4_)_2_S_2_O_8_ (1:1 volume ratio) at room temperature for 35 min. After removing the samples, they were cleaned with deionized water and dried with nitrogen gas. More details can be found somewhere else.^[^ ^41^ ^]^ Finally, the samples were immersed in the PFDTS alcohol solutions with a concentration of 1% for 2 h and dried in an oven at 90 °C for 60 min to achieve low‐surface‐energy modification. Both laser‐ablated microcone arrays and pristine copper surfaces were subjected to the identical chemical oxidation and low‐surface‐energy modification processes described above, thereby yielding nRS control surfaces and NWs surfaces, respectively. The detailed fabrication processes were shown in Table S1 (Supporting Information). To demonstrate the technological viability of our approach for practical engineering applications, the RS was also fabricated over a large area of 100 mm × 120 mm, as shown in Figure S1 (Supporting Information).

### Characterization and Measurement

The surface morphology of the samples was obtained by a field emission scanning electron microscope (SEM, TESCAN MIRA 3 LMH). The composition and distribution of surface elements were analyzed using an energy‐dispersive spectrometer (EDS, Oxford). The contact angles and sliding angles on the sample surfaces were measured by a video‐based optical contact angle measuring device (OCA 15 Plus from Data Physics Instruments). The adopted droplets were deionized water with a volume of 5 µL. The contact angles and sliding angles of every sample were tested at least three times at random locations to obtain average results.

### Evaporation Experiments

A 5 µL deionized water droplet was dripped onto the sample surface, which was then horizontally placed on the contact angle measuring device. The droplet evaporated naturally at room temperature. The volume change of the droplet during the evaporation process was recorded in real‐time using the OCA measuring device. After the experiment, image processing software was used to calculate the contact angle, TPCL diameter, and corresponding Laplace pressure.

### Delay Icing Experiments

Delay icing experiments were conducted in a low‐temperature freezer (Figure S10, Supporting Information). The internal humidity was adjusted using a humidifier with an accuracy of 5%. The minimum temperature of the freezer could reach −55 °C. The sample was placed into the freezer, and when the temperature and humidity stabilized at the set values, a 20 µL droplet was dripped on the sample surface. A CCD camera with an adjustable zoom lens, capturing 10 frames per second and a resolution of 0.2 µm, was used to record the top view of the droplet in real‐time. Simultaneously, the temperature changes of the droplet were monitored using a thermal infrared imager (FLUKE‐Ti480P).

### Static Anti‐Frosting Tests

The sample was placed on a Peltier cooling plate (TEC2‐19006) equipped with dual‐probe temperature recorders on both sides. The temperature recorders were used to record real‐time temperatures with a measuring accuracy of 0.1 °C. The water‐cooling device was installed beneath the cooling plate. The thermal conductive silicone grease (RG‐ICFN‐200G‐B1) was evenly smeared on the contact surfaces between the cooling plate and the sample, as well as between the cooling plate and the water‐cooling device. The ambient temperature and humidity were controlled at 15 ± 2 °C and 80 ± 5% RH, respectively. The cooling system was activated to gradually reduce the sample temperature from 15 °C to −15 °C, which was then maintained at −15 °C. Frost gradually began to form on the sample surface. A CCD camera with a frame rate of 10 and a resolution of 0.2 µm was used to record real‐time top and side views of the sample.

### Ice Adhesion Strength Measurement

The experimental setup for measuring ice adhesion strength was shown in Figure S32 (Supporting Information). First, the sample was placed into the freezer, and a cuvette was positioned on the sample surface. Then, 2 mL of water was added through the opening on top of the cuvette. The freezer was maintained at −20 °C and 20 ± 5% RH for 2 h to ensure complete freezing. Finally, a force transducer (Imada ZP‐100N) was used to push the ice column from the bottom of the cuvette at a speed of 0.5 mm s^−1^. The force‐displacement curve during ice detachment was recorded using computer software connected to the force transducer. The ice adhesion strength was calculated as the ratio of the peak force to the cross‐sectional area of the ice column. The force transducer had an accuracy of 0.01 N, and the cross‐sectional area of the ice column was 10 mm × 10 mm. The ice adhesion strength of each sample was measured at least three times.

### Experimental Observation of the Icing Process

The sample was fixed on the Peltier cooling plate using thermal conductive silicone grease. Dual‐probe temperature recorders on both sides of the cooling plate were used to record real‐time temperatures. The initial ambient temperature was 15 ± 2 °C, and the humidity around the sample was regulated using a humidifier. A 10 µL droplet was placed at the edge of the sample surface to facilitate side‐view observation of the icing phenomenon. After running the cooling system, the surface temperature of the sample gradually decreased from 15 °C to −15 °C or −40 °C, with cooling rates of 0.08 °C s^−1^ or 0.15 °C s^−1^, respectively. The cooling system was then maintained to keep the sample surface temperature at the set minimum value. The total time for cooling and maintaining a constant temperature was fixed at 440 s. Similarly, a CCD camera with 10 frames per second and a resolution of 0.2 µm was used to record real‐time side views of the droplet. The droplet penetration depth and the remaining air pocket volume were obtained using image processing software.

### Icing and Melting Cycle Experiments

The icing and melting cycle experiments were conducted on the aforementioned Peltier cooling plate. The ambient temperature and humidity were maintained at 15 ± 2 °C and 80 ± 5% RH, respectively. The droplet volume was fixed at 5 µL. After activating the cooling system, the surface temperature of the sample gradually decreased from 15 °C to −15 °C. When the temperature reached −15 °C, the cooling system was turned off, allowing the sample temperature to naturally rise back to 15 °C. A horizontally positioned CCD camera was used to record real‐time side views of the droplet during the icing and melting processes. The standard deviation of the measured contact angles is less than 3° for CA ≥ 150° and ≈5° for CA ≤ 140°. The uncertainty in TPCL diameter evaluation is 0.002‐0.03 mm, with an average standard deviation of 0.02 mm.

## Conflict of Interest

The authors declare no conflict of interest.

## Supporting information



Supporting Information

Supplemental Video 1

Supplemental Video 1

Supplemental Video 1

Supplemental Video 1

## Data Availability

The data that support the findings of this study are available from the corresponding author upon reasonable request.
