# Supplementary material for: Re‐Entrant Super‐Repellent Metallic Structures for Robust Anti‐Icing
Source: Adv Sci (Weinh). 2025 Aug 19;12(39):e08272. doi: 10.1002/advs.202508272 (PMC12533129; doi:10.1002/advs.202508272)
Supplement: Supplementary file 1 — Supporting Information [file ADVS-12-e08272-s003.docx]

Supporting Information

Re-entrant Super-Repellent Metallic Structures for Robust Anti-Icing

*Daizhou Li^1^, Lizhong Wang^1^, Rui Peng^1^, Ziyan Song^1^, Zhao Liu^1^, Zhixuan Chang^1^, Hongjun Zhang^1^, Peixun Fan^1^* and Minlin Zhong^1^**

Laser Materials Processing Research Center

Key Laboratory for Advanced Materials Processing Technology (Ministry of Education)

Joint Research Center for Advanced Materials & Anti-icing of Tsinghua University (SMSE)-AVIC ARI

School of Materials Science and Engineering

Tsinghua University

Beijing 100084, P. R. China

E-mail: fanpeixun@tsinghua.edu.cn, zhml@tsinghua.edu.cn

**Contents:**

1. **Supplementary Discussion**
   1. Calculation of breakthrough pressure for wetting transition
   2. Calculation of air pocket pressure for nRS and RS
   3. Thermodynamic analysis and energy barrier calculation for droplet wetting process
2. **Supplementary Table: Table S1**
3. **Supplementary Figures: Figure S1-S32**
4. **Supplementary Videos: Video S1-S4**

# Supplementary Discussion

1. Calculation of breakthrough pressure for wetting transition

Under static conditions, the contact states of the droplet with the two structures are illustrated in Figure S5. Considering a single structural unit on the surface, the vertical component of the surface tension provided by the contact line between the droplet and the structure balances the Laplace pressure difference across the meniscus. The equilibrium condition in the *z*-direction can be expressed as:

$\gamma\int_{l} sin\theta_{lv}ds=\Delta p\int_{S} cos\theta_{p}dA$ (S1)

where $\Delta p$ represents the pressure difference across the liquid-air interface; *γ* denotes the surface tension of the liquid; $\theta_{\mathrm{lv}}$ is the angle formed by the liquid-air surface tension normal to the boundary and the *xy*-plane; $\theta_{p}$ is the angle between the normal vector of the liquid-air interface and the *z*-direction. When $\theta_{\mathrm{lv}}$ is a constant, Equation S1 can be transformed as follows:

$\Delta p=\frac{\gamma lsin\theta_{\mathrm{lv}}}{A_{p}}$ (S2)

where $l$ denotes the length of the liquid-solid contact line; $A_{p}$ represents the projected area of the liquid-air interface on the *xy*-plane.

According to the geometric relationship shown in Figure S5, when $\theta_{\mathrm{lv}}=\theta_{\mathrm{adv}}-\frac{\pi}{2}-\frac{\alpha}{2}$, the breakthrough pressure $\Delta p$ of nRS reaches its maximum value, which can be expressed as:

$\Delta p_{\max}=\gamma\frac{2\pi h_{1}\tan\frac{\alpha}{2}}{P^{2}-\pi h_{1}^{2}{tan}^{2}\frac{\alpha}{2}}\sin(\theta_{\mathrm{adv}}-\frac{\pi}{2}-\frac{\alpha}{2})$ (S3)

It is well known that RSs can not only repel water but also resist the intrusion of liquids with lower surface tension. When a low surface tension liquid with $\theta_{\mathrm{adv}}\leq\frac{\pi}{2}$ is applied, the value of the breakthrough pressure depends on the magnitude of $\sin{(\theta}_{\mathrm{adv}})$. When $\theta_{\mathrm{adv}}>\frac{\pi}{2}$, the shape of the liquid-air interface sags into a semicircular form, and at this point, the interface intersects the contact line perpendicularly. The breakthrough pressure $\Delta p$ of RS reaches its maximum value, which can be expressed as:

${\Delta p}_{\max}= \left\{ \begin{aligned} \gamma\frac{4\pi D}{4P^{2}-\pi D^{2}}\sin\left( \theta_{\mathrm{adv}} \right) (\theta_{\mathrm{adv}}\leq\frac{\pi}{2}) \\ \gamma\frac{4\pi D}{4P^{2}-\pi D^{2}} (\theta_{\mathrm{adv}}>\frac{\pi}{2}) \end{aligned} \right.$ (S4)

Here, $\theta_{\mathrm{adv}}$ represents the apparent advancing contact angle of the liquid on the sidewall of the microstructure; $h_{1}$ and *α* denote the height of the triple-phase contact line (TPCL) descent and the taper angle of nRS, respectively; *P* is the center-to-center spacing of the micro-scale T-shaped array; *D* is the cap diameter of RS.

1. Calculation of air pocket pressure for nRS and RS

Both nRS and RS are open structures that allow free circulation of gas. As the temperature decreases, the gas in the air pockets gradually dissolves into the supercooled liquid droplet, leading to a reduction in the amount of gas in the structure. Thanks to the connectivity of both structures to the atmospheric environment, the gas lost due to increased solubility can be rapidly replenished by the surrounding air, thereby maintaining the stability of the gas amount in the air pockets. By analyzing the energy changes at the liquid-air-solid triple-phase interface and treating the gas in the air pockets as an ideal gas, the general form of the air pocket pressure calculation model for RS and nRS can be expressed as:

$P_{\mathrm{app}}=P_{0}\ln\frac{V_{0}^{\mathrm{eff}}}{V_{0}^{\mathrm{eff}}-{(V_{m}^{\mathrm{eff}})}_{\max}}$ (S5)

where $P_{\mathrm{app}}$ represents the pressure of the trapped air pockets in the structure; $P_{0}$ denotes the ambient atmospheric pressure. $P_{0}$ varies with changes in the ambient temperature and can be expressed as: $P_{0}=1.04-2.69\times{10}^{-12}e^{T/{1.44}}-0.016e^{T/{37.29}}$ (*T* is the ambient temperature). $V_{0}^{\mathrm{eff}}$ is the effective geometric volume of the air pocket in the initial state, and $V_{m}^{\mathrm{eff}}$ is the effective geometric volume of the meniscus.

In a closed system, there is no need to consider the exchange of energy and matter with the external environment. To facilitate subsequent model derivation, we equivalently transform the open structure into a corresponding closed structure. According to the equivalent method proposed by Xue et al.,^[1]^ the pillar structure can be approximately equivalent to a pore structure. The corresponding effective geometric radius and capillary radius can be expressed as:

$\left\{ \begin{aligned} r_{g}^{\mathrm{eff}}=(\sqrt{\frac{\pi}{2f_{p}}}-1)r_{p} \\ \\ r_{c}^{\mathrm{eff}}=\frac{1-f_{p}}{f_{p}}r_{p} \end{aligned} \right.$ (S6)

where $r_{p}$ represents the radius of the pillar structure; $f_{p}$ is the area fraction of the pillar structure when equivalently transformed into a pore structure.

For nRS surface, according to Figure S5 and Equation S6, $V_{0}^{\mathrm{eff}}$ and ${(V_{m}^{\mathrm{eff}})}_{\max}$ can be expressed as follows:

$V_{0}^{\mathrm{eff}}=\frac{1}{3}\pi({r_{0}^{\mathrm{eff}})}^{2}H$ (S7)

${(V_{m}^{\mathrm{eff}})}_{\max}=\frac{1}{3}\pi[({r_{0}^{\mathrm{eff}})}^{2}+({r_{a}^{\mathrm{eff}})}^{2}+r_{0}^{\mathrm{eff}}\cdot r_{a}^{\mathrm{eff}}]\cdot{{(H}_{a}^{\mathrm{eff}})}_{\max}$ (S8)

$r_{a}^{\mathrm{eff}}=r_{0}^{\mathrm{eff}}-{{(H}_{a}^{\mathrm{eff}})}_{\max}\cdot\tan\frac{\alpha}{2}$ (S9)

$r_{0}^{\mathrm{eff}}=(\sqrt{\frac{\pi}{2f_{1}}}-1)r_{0}$ (S10)

${{(H}_{a}^{\mathrm{eff}})}_{\max}=\frac{f_{1}}{1-f_{1}}{(\sqrt{\frac{\pi}{2f_{1}}}-1)}^{3}({H_{a})}_{\max}$ (S11)

$H_{a}=\frac{r_{0}[2-3sin\left( \theta-\frac{\alpha}{2} \right)+{sin}^{3}\left( \theta-\frac{\alpha}{2} \right)]}{-3{cos}^{3}(\theta-\frac{\alpha}{2})}$ (S12)

The protruding meniscus is equivalently modeled as a truncated cone. Since $\alpha$ is small, to simplify the calculation, it can be assumed that $\tan\frac{\alpha}{2}\approx0$, . Under this condition, the pressure of the air pocket can be expressed as:

$P_{\mathrm{app}}=P_{0}\ln\frac{H}{H-{(H_{a}^{\mathrm{eff}})}_{\max}}$ (S13)

Where $\theta$ is the sag angle of the liquid-air interface. When $\theta=\theta_{\mathrm{adv}}$, $H_{a}$ reaches its maximum value $({H_{a})}_{\max}$. Due to the small contact angle hysteresis of the micro-nano hierarchical structure, the apparent contact angle of the nanostructure $\theta_{ca2}$ is set equal to the advancing angle $\theta_{\mathrm{adv}}$. It can be expressed as: $\cos\theta_{\mathrm{adv}}=f_{2}\left( 1+\cos\theta_{Y} \right)-1$. $f_{1}$ and $f_{2}$ are the area fractions of the microstructures and nanostructures, respectively; *H* is the height of the microstructures; $r_{0}$ and $r_{a}$ are the radius at the top of the structures and the radius of the structures corresponding to the base of the liquid truncated cone, respectively; $H_{a}^{\mathrm{eff}}$ is the effective value of the average meniscus height $H_{a}$; $\theta_{Y}$ is the contact angle of the liquid on a smooth sidewall.

Owing to the presence of a unique reentrant feature, the derivation process of the air pocket pressure calculation model for RS is as follows:

$V_{0}^{\mathrm{eff}}=\frac{\pi}{4}(D^{\mathrm{eff}})^{2}h+\frac{\pi}{4}({d^{\mathrm{eff}})}^{2}(H-h)$ (S14)

${(V_{m}^{\mathrm{eff}})}_{\max}=\frac{\pi}{4}({D^{\mathrm{eff}})}^{2}\cdot{{(H}_{a}^{\mathrm{eff}})}_{\max}$ (S15)

${{(H}_{a}^{\mathrm{eff}})}_{\max}=\frac{f_{1}}{1-f_{1}}{(\sqrt{\frac{\pi}{2f_{1}}}-1)}^{3}({H_{a})}_{\max}$ (S16)

$({H_{a})}_{\max}=\frac{\frac{D}{2}[2-3sin\theta_{\mathrm{adv}}+{sin}^{3}\theta_{\mathrm{adv}}]}{-3{cos}^{3}\theta_{\mathrm{adv}}}$ (S17)

$D^{\mathrm{eff}}=(\sqrt{\frac{\pi}{2f_{1}}}-1)D$ (S18)

$d^{\mathrm{eff}}=(\sqrt{\frac{\pi}{2f_{1a}}}-1)d$ (S19)

$\sigma^{2}=\frac{{{(d}^{\mathrm{eff}})}^{2}}{({D^{\mathrm{eff}})}^{2}}$ (S20)

When the droplet is pinned at the lower corner of the microstructure cap and assumes a hemispherical shape, the air pocket state of RS is most stable, and the air pocket pressure can be calculated as:

$P_{\mathrm{app}}=P_{0}\ln\frac{h+\sigma^{2}(H-h)}{\sigma^{2}(H-h)-{(H_{a}^{\mathrm{eff}})}_{\max}}$ (S21)

Here, *d* and *h* represent the pillar diameter and cap thickness of RS, respectively; $f_{1a}$ denotes the area fraction of straight micropillars with the same pillar diameter and pillar spacing as RS, expressed as $f_{1a}=\frac{\pi d^{2}}{4P^{2}}$; $\sigma^{2}$ represents the squared ratio of the effective geometric radii corresponding to the pillar diameter and the cap diameter.

1. Thermodynamic analysis and energy barrier calculation for droplet wetting process

To elucidate the intrinsic mechanisms by which different structures maintain the stability of the CB state, we analyzed the thermodynamic energy changes during the wetting process of droplets on two types of surfaces. Figure S25 illustrates the gradual penetration processes of droplets on nRS and RS surfaces. We assume that the entire solid-liquid-air system is an isothermal system, the wetting process is reversible, and the gas obeys the ideal gas law. Adopting the corresponding closed structure for analysis, the free energy of the entire system can be expressed as:^[2]^

$G_{T}=\gamma_{\mathrm{LV}}S_{\mathrm{LV}}+\gamma_{\mathrm{SL}}S_{\mathrm{SL}}+\gamma_{\mathrm{SV}}S_{\mathrm{SV}}+G_{\mathrm{air}}$ (S22)

$G_{\mathrm{air}}=G_{P_{0}}+nRT\cdot ln\frac{V_{0}}{V_{1}}$ (S23)

Here, $G_{T}$ represents the free energy of the system; $G_{\mathrm{air}}$ denotes the energy of the gas within the air pocket; $G_{P_{0}}$ represents the free energy of the gas in its initial state within the air pocket; $\gamma_{\mathrm{LV}}$, $\gamma_{\mathrm{SL}}$​, and $\gamma_{\mathrm{SV}}$ represent the liquid-vapor, solid-liquid, and solid-vapor surface tensions, respectively; $S_{\mathrm{LV}}$, $S_{\mathrm{SL}}$, and $S_{\mathrm{SV}}$ are the areas of the liquid-vapor, solid-liquid, and solid-vapor interfaces, respectively; The initial pressure and temperature of the ambient gas are $P_{0}$ and *T*, respectively; *n* is the amount of substance of the gas; *R* is the ideal gas constant; $V_{0}$ and $V_{1}$ represent the volumes of the air pocket in the initial and final states, respectively.

We assume that the free energy of the system in its initial state is $G_{0}$, the free energy of the system in its current state after transition can be expressed as:

$G_{T}=G_{0}+\gamma_{\mathrm{LV}}\Delta S_{\mathrm{LV}}+\gamma_{\mathrm{SL}}\Delta S_{\mathrm{SL}}+\gamma_{\mathrm{SV}}{\Delta S}_{\mathrm{SV}}+nRT\cdot\ln\frac{V_{0}}{V_{1}}$ (S24)

where $\Delta S_{\mathrm{LV}}$, $\Delta S_{\mathrm{SL}}$ and ${\Delta S}_{\mathrm{SV}}$ represent the changes in the areas of the liquid-vapor, solid-liquid, and solid-vapor interfaces, respectively. First, we analyze the wetting process of the droplet on nRS surface. Based on the results shown in Figure S25, the wetting transition of the droplet on nRS surface is divided into three stages: (1) TPCL remains pinned at the edge while the liquid-air interface sags downward; (2) TPCL slides downward along the sidewall of the structure until it contacts the bottom of the cavity; (3) the gas dissolves into the droplet, and the microstructure becomes fully wetted. During stage (1), TPCL is pinned at the edge of the microstructure, and the meniscus gradually sags into the interior of the structure. At this point, the droplet is in the CB state, corresponding to the equivalent pore structure, and the free energy of the system can be expressed as:

$G_{1}=G_{0}+N\pi r_{0}^{2}\gamma_{\mathrm{LV}}\cdot\left[ \frac{1-sin(\theta-\frac{\alpha}{2})}{1+sin(\theta-\frac{\alpha}{2})}+\frac{P_{0}H}{{3\gamma}_{\mathrm{LV}}}\ln\left( \frac{H}{H-H_{a1}} \right) \right]$ (S25)

$H_{a1}=\frac{r_{0}[2-3sin(\theta-\frac{\alpha}{2})+{sin}^{3}(\theta-\frac{\alpha}{2})]}{-3{cos}^{3}(\theta-\frac{\alpha}{2})}$ (S26)

where *N* represents the number of structural units beneath the droplet; $H_{a1}$ is the average protrusion depth of the droplet at the top of the microstructure

As the liquid-air interface continues to sag, the sag angle *θ* increases continuously. When $\theta=\theta_{\mathrm{adv}}$, TPCL begins to move downward, and the wetting process enters the second stage. At this point, the droplet transitions from the CB state to the Wenzel state. The free energy of the system is related to the downward movement depth $h_{1}$ of the TPCL and can be expressed as:

$G_{2}=G_{0}+N\pi r_{0}^{2}\gamma_{\mathrm{LV}}\cdot\left[ \frac{2r_{a}^{2}}{(1+\sin\left( \theta_{\mathrm{adv}}-\frac{\alpha}{2} \right))r_{0}^{2}}-1-(\frac{h_{1}r_{a}+h_{1}r_{0}}{r_{0}^{2}\cos\frac{\alpha}{2}})\left( f_{2}cos\theta_{Y}+f_{2}-1 \right)+\frac{P_{0}H}{{3\gamma}_{\mathrm{LV}}}\ln\left( \frac{r_{0}^{2}H}{r_{a}^{2}(H-h_{1}-\left( H_{a2} \right)_{\max}} \right) \right]$ (S27)

$r_{a}=r_{0}-h_{1}\tan\frac{\alpha}{2}$ (S28)

where $r_{a}$ represents the radius of the pore structure corresponding to the downward movement $h_{1}$ of TPCL, and $H_{a2}$ denotes the average protrusion depth of the droplet at $r_{a}$. During the third stage of the wetting process, when the sagging liquid-air interface contacts the bottom of the structure, the gas within the air pocket gradually dissolves into the droplet, and the microstructure is completely wetted. At this point, the droplet is in the Wenzel state, and the free energy of the system can be expressed as:

$G_{3}=G_{0}-N\pi r_{0}^{2}\gamma_{\mathrm{LV}}\cdot\left[ 1+\left( \frac{H}{r_{0}cos\frac{\alpha}{2}} \right)\cdot\left( f_{2}cos\theta_{Y}+f_{2}-1 \right) \right]-G_{P_{0}}$ (S29)

To present the free energy equation of the system more clearly and intuitively, we define the nondimensionalized free energy formula as follows:

$G_{T}^{*}=\frac{\left( G-G_{0} \right)\left( 1-f_{1} \right)}{N\pi r_{0}^{2}\gamma_{\mathrm{LV}}}$ (S30)

By combining Equation S6 and Equations S25–S30, the nondimensionalized total energy during the transition of the droplet from the CB state to the Wenzel state can be calculated according to the following formula:

$G_{T}^{*}=\left\{ \begin{aligned} \left( 1-f_{1} \right)\cdot\left[ \frac{1-sin(\theta-\frac{\alpha}{2})}{1+sin(\theta-\frac{\alpha}{2})}+\frac{P_{0}H}{{3\gamma}_{\mathrm{LV}}}\ln\left( \frac{H}{H-H_{a1}^{\mathrm{eff}}} \right) \right], \left( \frac{\pi}{2}\leq\theta<\theta_{\mathrm{adv}} \right) \\ \\ \\ \left( 1-f_{1} \right)\cdot\left[ \frac{2{{(r}_{a}^{\mathrm{eff}})}^{2}}{(1+\sin\left( \theta_{\mathrm{adv}}-\frac{\alpha}{2} \right))({r_{0}^{\mathrm{eff}})}^{2}}-1-(\frac{h_{1}r_{a}^{\mathrm{effc}}+h_{1}r_{0}^{\mathrm{effc}}}{({r_{0}^{\mathrm{effc}})}^{2}\cos\frac{\alpha}{2}})\left( f_{2}cos\theta_{Y}+f_{2}-1 \right)+\frac{P_{0}H}{{3\gamma}_{\mathrm{LV}}}\ln\left( \frac{({r_{0}^{\mathrm{eff}})}^{2}H}{{{(r}_{a}^{\mathrm{eff}})}^{2}(H-h_{1}-{{(H}_{a2}^{\mathrm{eff}})}_{\max})} \right) \right], \\ \left( \theta=\theta_{\mathrm{adv}}, 0\leq h_{1}\leq H-{{(H}_{m}^{\mathrm{eff}})}_{\max} \right) \\ \\ \\ -\left( 1-f_{1} \right)-\left( 1-f_{1} \right)\left( \frac{H}{r_{0}^{\mathrm{effc}}cos\frac{\alpha}{2}} \right)\cdot\left( f_{2}cos\theta_{Y}+f_{2}-1 \right)-\frac{P_{0}H\left( 1-f_{1} \right)}{3\gamma_{\mathrm{LV}}}, \left( h_{1}=H \right) \end{aligned} \right.$(S31)

where $G_{T}^{*}$ represents the nondimensionalized free energy of the system; $H_{m}^{\mathrm{eff}}$ represents the equivalent value of the meniscus height $H_{m}$, expressed as $H_{m}^{\mathrm{eff}}=(\sqrt{\frac{\pi}{2f_{1a}}}-1)H_{m}$, $H_{m}=r_{\mathrm{pa}}\cdot\frac{sin(\theta-\frac{\alpha}{2})-1}{cos(\theta-\frac{\alpha}{2})}$; When $\theta=\theta_{\mathrm{adv}}$, $H_{m}$ reaches its maximum value $({H_{m})}_{\max}$; At this point, $H_{m}^{\mathrm{eff}}$ also attains its maximum value ${{(H}_{m}^{\mathrm{eff}})}_{\max}$. $r_{p}$ and $r_{\mathrm{pa}}$ represent the top radius of nRS and the radius of nRS corresponding to the downward movement $h_{1}$ of TPCL, respectively; $r_{0}^{\mathrm{eff}}$ and $r_{0}^{\mathrm{effc}}$ denote the effective geometric radius and effective capillary radius of $r_{p}$, respectively; $r_{a}^{\mathrm{eff}}$ and $r_{a}^{\mathrm{effc}}$ represent the effective geometric radius and effective capillary radius of $r_{\mathrm{pa}}$, respectively; $H_{a1}^{\mathrm{eff}}$ and $H_{a2}^{\mathrm{eff}}$ are the equivalent values of the average protrusion depth of the droplet at $r_{p}$ and $r_{\mathrm{pa}}$, respectively; and $f_{1a}$ is the area fraction occupied by straight micropillars with a radius of $r_{\mathrm{pa}}$ and a pillar spacing of *P*, given by $f_{1a}=\frac{\pi r_{\mathrm{pa}}^{2}}{P^{2}}$. The equations for calculating the relevant parameters are as follows:

$r_{0}^{\mathrm{eff}}=(\sqrt{\frac{\pi}{2f_{1}}}-1)r_{p}$ (S32)

$r_{0}^{\mathrm{effc}}=\frac{1-f_{1}}{f_{1}}r_{p}$ (S33)

$r_{a}^{\mathrm{eff}}=(\sqrt{\frac{\pi}{2f_{1a}}}-1)r_{\mathrm{pa}}$ (S34)

$r_{a}^{\mathrm{effc}}=\frac{1-f_{1a}}{f_{1a}}r_{\mathrm{pa}}$ (S35)

$({H_{a2})}_{\max}=\frac{r_{\mathrm{pa}}[2-3sin\left( \theta_{\mathrm{adv}}-\frac{\alpha}{2} \right)+{sin}^{3}\left( \theta_{\mathrm{adv}}-\frac{\alpha}{2} \right)]}{-3{cos}^{3}(\theta_{\mathrm{adv}}-\frac{\alpha}{2})}$ (S36)

${{(H}_{a2}^{\mathrm{eff}})}_{\max}=\frac{f_{1a}}{1-f_{1a}}{(\sqrt{\frac{\pi}{2f_{1a}}}-1)}^{3}({H_{a2})}_{\max}$ (S37)

When $h_{1}=H-{{(H}_{m}^{\mathrm{eff}})}_{\max}$, the droplet penetrates to the bottom of the microstructure. Let $\beta=\frac{sin(\theta_{\mathrm{adv}}-\frac{\alpha}{2})-1}{cos(\theta_{\mathrm{adv}}-\frac{\alpha}{2})}$, then ${{(H}_{m}^{\mathrm{eff}})}_{\max}=r_{a}^{\mathrm{eff}}\cdot\beta$. We can derive the formula for calculating $r_{\mathrm{pa}}$ as follows:

$r_{\mathrm{pa}}=\frac{r_{p}+Htan\frac{\alpha}{2}-\frac{\sqrt{2}}{2}P\beta\cdot tan\frac{\alpha}{2}}{1-\beta\cdot tan\frac{\alpha}{2}}$ (S38)

It can be observed that the energy barrier hindering the transition of the droplet from the CB state to the Wenzel state consists of three components: the meniscus energy barrier caused by the pinning of TPCL, the capillary energy barrier associated with the downward movement of TPCL, and the energy barrier due to the compression of the gas within the air pocket. Therefore, the nondimensionalized total energy barrier of nRS surface during the wetting process can be expressed as:

${\Delta G}_{\mathrm{nRS}}^{*}=\left( 1-f_{1} \right)\cdot\left[ \frac{2{{(r}_{a}^{\mathrm{eff}})}^{2}}{(1+\sin\left( \theta_{\mathrm{adv}}-\frac{\alpha}{2} \right))({r_{0}^{\mathrm{eff}})}^{2}}-1-(\frac{r_{a}^{\mathrm{effc}}+r_{0}^{\mathrm{effc}}}{({r_{0}^{\mathrm{effc}})}^{2}\cos\frac{\alpha}{2}})(H-{{(H}_{m}^{\mathrm{eff}})}_{\max})\left( f_{2}cos\theta_{Y}+f_{2}-1 \right)+\frac{P_{0}H}{{3\gamma}_{\mathrm{LV}}}\ln\left( \frac{({r_{0}^{\mathrm{eff}})}^{2}H}{{{(r}_{a}^{\mathrm{eff}})}^{2}({{(H}_{m}^{\mathrm{eff}})}_{\max}-{{(H}_{a2}^{\mathrm{eff}})}_{\max})} \right) \right]$ (S39)

To correspond to the cap barrier of RS, we also calculate the nondimensionalized barrier ${\Delta G}_{h}^{*}$ when TPCL moves downward by a depth *h*:

${\Delta G}_{h}^{*}=\left( 1-f_{1} \right)\cdot\left[ \frac{2{{(r}_{a}^{\mathrm{eff}})}^{2}}{(1+\sin\left( \theta_{\mathrm{adv}}-\frac{\alpha}{2} \right))({r_{0}^{\mathrm{eff}})}^{2}}-1-\left( \frac{hr_{a}^{\mathrm{effc}}+hr_{0}^{\mathrm{effc}}}{({r_{0}^{\mathrm{effc}})}^{2}\cos\frac{\alpha}{2}} \right)\left( f_{2}cos\theta_{Y}+f_{2}-1 \right)+\frac{P_{0}H}{{3\gamma}_{\mathrm{LV}}}\ln\left( \frac{({r_{0}^{\mathrm{eff}})}^{2}H}{{{(r}_{a}^{\mathrm{eff}})}^{2}(H-h-\left( H_{a2}^{\mathrm{eff}} \right)_{\max}} \right) \right]$ (S40)

Here, *h* represents the cap thickness of RS. When $h_{1}=h$, $r_{\mathrm{pa}}=r_{p}+h\tan\frac{\alpha}{2}$. For nRS, $r_{p}=15 \mu m$, $\alpha=20^{\circ}$, and $f_{1}=0.022$.

Due to the curvature variation in the vertical direction, the wetting process of the droplet on RS surface is more complex. We summarize the entire penetration process on RS surface into five stages, as illustrated in Figure S25. The first stage similarly involves the pinning of TPCL and the protrusion of the liquid-air interface. The free energy of the system during this stage can be expressed as:

$G_{1}=G_{0}+N\pi r^{2}\gamma_{\mathrm{LV}}\cdot\left[ \frac{1-sin\theta}{1+sin\theta}+\frac{P_{0}H_{V}}{\gamma_{\mathrm{LV}}}\ln\left( \frac{H_{V}}{H_{V}-H_{a1}} \right) \right]$ (S41)

where *r* is the top radius of the pore structure corresponding to RS; $H_{V}$ is the ratio of the initial stored air volume to the top projected area, expressed as $H_{V}= h+\frac{R^{2}}{r^{2}}\left( H-h \right)$; $H_{a1}$ is the average protrusion depth of the droplet when the radius of the pore structure is *r*, given by $H_{a1}=\frac{r[2-3sin\theta+{sin}^{3}\theta]}{-3{cos}^{3}\theta}$. When the sag angle of the liquid-air interface reaches the apparent advancing angle, TPCL moves downward until it is pinned at the lower corner of RS cap. This marks the second stage of the wetting process. The free energy of the system can be expressed as:

$G_{2}=G_{0}+N\pi r^{2}\gamma_{\mathrm{LV}}\cdot\left[ \frac{1-sin\theta}{1+sin\theta}-\frac{2h_{1}}{r}\left( f_{2}cos\theta_{Y}+f_{2}-1 \right)+\frac{P_{0}H_{V}}{\gamma_{\mathrm{LV}}}\ln\left( \frac{H_{V}}{H_{V}-{{(H}_{a1})}_{\max}-h_{1}} \right) \right]$ (S42)

During the third stage of the wetting process, the droplet pinned at the lower corner of RS cap continues to expand until it contacts the sidewall of the pillar beneath the cap. The free energy of the system can be expressed as:

$G_{3}=G_{0}+N\pi r^{2}\gamma_{\mathrm{LV}}\cdot\left[ \frac{1-cos\theta_{1}}{1+cos\theta_{1}}-\frac{2h}{r}\left( f_{2}cos\theta_{Y}+f_{2}-1 \right)+\frac{P_{0}H_{V}}{\gamma_{\mathrm{LV}}}\ln\left( \frac{H_{V}}{H_{V}-H_{a2}-h} \right) \right]$ (S43)

where $\theta_{1}$ represents the angle formed between the liquid-air surface tension and the *xy*-plane, given by $\theta_{1}=\theta-\frac{\pi}{2}$; $H_{a2}$ denotes the average protrusion depth of the droplet when TPCL moves downward by *h*, expressed as $H_{a2}=\frac{r[2-3cos\theta_{1}+{cos}^{3}\theta_{1}]}{3{sin}^{3}\theta_{1}}$​. After the droplet contacts the sidewall of the pillar, TPCL continues to move downward from this position, and the free energy of the system can be expressed as:

$G_{4}=G_{0}+N\pi r^{2}\gamma_{\mathrm{LV}}\cdot\left[ \frac{2{R^{2}}/{r^{2}}}{1+sin\theta_{\mathrm{adv}}}-1-(\frac{2h}{r}+\frac{2R}{r^{2}}(h_{1}-h))\left( f_{2}cos\theta_{Y}+f_{2}-1 \right)+\frac{P_{0}H_{V}}{\gamma_{\mathrm{LV}}}\ln\left( \frac{H_{V}}{H_{V}-h-({R^{2}}/{r^{2}})({{(H}_{a3})}_{\max}+h_{1}-h)} \right) \right]$ (S44)

where *R* is the geometric radius of the pore structure corresponding to the pillar radius of RS; $H_{a3}$ is the average protrusion depth of the droplet when the radius of the pore structure is *R*, given by $H_{a3}=\frac{R[2-3sin\theta+{sin}^{3}\theta]}{-3{cos}^{3}\theta}$. In the final stage of the penetration process, the meniscus contacts the bottom of the structure, and the gas completely dissolves into the droplet. The droplet on the microstructure undergoes a wetting transition from the CB state to the Wenzel state. At this point, the free energy of the system can be expressed as:

$G_{5}=G_{0}-N\pi r^{2}\gamma_{\mathrm{LV}}\cdot\left[ 1+\left( \frac{2h}{r}+\frac{2R(H-h)}{r^{2}}+\frac{R^{2}}{r^{2}} \right)\cdot\left( f_{2}cos\theta_{Y}+f_{2}-1 \right) \right]-G_{P_{0}}$ (S45)

Similarly, the nondimensionalized free energy $G_{T}^{*}$ for the wetting process on RS surface is calculated as $G_{T}^{*}=\frac{\left( G-G_{0} \right)\left( 1-f_{1} \right)}{N\pi r^{2}\gamma_{\mathrm{LV}}}$. By combining Equation S6 and Equations S41–S45, the formula for calculating the nondimensionalized free energy of the system can be derived as follows:

$G_{T}^{*}=\left\{ \begin{aligned} \left( 1-f_{1} \right)\cdot\left[ \frac{1-sin\theta}{1+sin\theta}+\frac{P_{0}H_{V}^{\mathrm{eff}}}{\gamma_{\mathrm{LV}}}\ln\left( \frac{H_{V}^{\mathrm{eff}}}{H_{V}^{\mathrm{eff}}-H_{a1}^{\mathrm{eff}}} \right) \right], \left( \frac{\pi}{2}\leq\theta<\theta_{\mathrm{adv}} \right) \\ \\ \\ \left( 1-f_{1} \right)\cdot\left[ \frac{1-sin\theta}{1+sin\theta}-\frac{2h_{1}}{r_{c}^{\mathrm{eff}}}\left( f_{2}cos\theta_{Y}+f_{2}-1 \right)+\frac{P_{0}H_{V}^{\mathrm{eff}}}{\gamma_{\mathrm{LV}}}\ln\left( \frac{H_{V}^{\mathrm{eff}}}{H_{V}^{\mathrm{eff}}-({H_{a1}^{\mathrm{eff}})}_{\max}-h_{1}} \right) \right], \\ \left( \theta=\theta_{\mathrm{adv}}, 0\leq h_{1}<h \right) \\ \\ \\ \left( 1-f_{1} \right)\cdot\left[ \frac{1-cos\theta_{1}}{1+cos\theta_{1}}-\frac{2h}{r_{c}^{\mathrm{eff}}}\left( f_{2}cos\theta_{Y}+f_{2}-1 \right)+\frac{P_{0}H_{V}^{\mathrm{eff}}}{\gamma_{\mathrm{LV}}}\ln\left( \frac{H_{V}^{\mathrm{eff}}}{H_{V}^{\mathrm{eff}}-H_{a2}^{\mathrm{eff}}-h} \right) \right] \\ h_{1}=h, \theta_{\mathrm{adv}}-\frac{\pi}{2}{\leq\theta}_{1}\leq\frac{\pi}{2}+arccos\theta_{2}, cos\theta_{2}=\frac{r}{R} \\ \\ \\ \left( 1-f_{1} \right)\cdot\left[ \begin{aligned} \frac{2\mu^{2}}{1+sin\theta_{\mathrm{adv}}}-1-\left( \frac{2h}{r_{c}^{\mathrm{eff}}}+\frac{2R_{c}^{\mathrm{eff}}}{\left( r_{c}^{\mathrm{eff}} \right)^{2}}\left( h_{1}-h \right) \right)\left( f_{2}cos\theta_{Y}+f_{2}-1 \right)+\frac{P_{0}H_{V}^{\mathrm{eff}}}{\gamma_{\mathrm{LV}}} \\ \ln\left( \frac{H_{V}^{\mathrm{eff}}}{H_{V}^{\mathrm{eff}}-h-\mu^{2}({{(H}_{a3}^{\mathrm{eff}})}_{\max}+h_{1}-h)} \right) \end{aligned} \right] \\ H_{1}<h_{1}\leq H-{{(H}_{m}^{\mathrm{eff}})}_{\max}, H_{1}=h+r\cdot tan\theta_{2} \\ \\ \\ -\left( 1-f_{1} \right)-\left( 1-f_{1} \right)\left( \frac{2h}{r_{c}^{\mathrm{eff}}}+\frac{2R_{c}^{\mathrm{eff}}(H-h)}{\left( r_{c}^{\mathrm{eff}} \right)^{2}}+\mu^{2} \right)\cdot\left( f_{2}cos\theta_{Y}+f_{2}-1 \right)-\frac{P_{0}H_{V}^{\mathrm{eff}}\left( 1-f_{1} \right)}{\gamma_{\mathrm{LV}}}, \left( h_{1}=H \right) \end{aligned} \right.$(S46)

Here, ${{(H}_{m}^{\mathrm{eff}})}_{\max}$ represents the maximum value of the equivalent meniscus height, given by ${{(H}_{m}^{\mathrm{eff}})}_{\max}=R^{\mathrm{eff}}\cdot\frac{sin\theta_{\mathrm{adv}}-1}{cos\theta_{\mathrm{adv}}}$; $r_{c}^{\mathrm{eff}}$ and $R_{c}^{\mathrm{eff}}$ denote the effective capillary radii corresponding to *D* and *d*, respectively; $r^{\mathrm{eff}}$ and $R^{\mathrm{eff}}$ represent the effective geometric radii corresponding to *D* and *d*, respectively; $H_{V}^{\mathrm{eff}}$ is the equivalent value of $H_{V}$, expressed as $H_{V}^{\mathrm{eff}}= h+\mu^{2}\left( H-h \right)$; $\mu^{2}$ is the squared ratio of the effective geometric radii corresponding to the pillar radius and the cap radius of RS, given by $\mu^{2}=\frac{{{(R}^{\mathrm{eff}})}^{2}}{({r^{\mathrm{eff}})}^{2}}$; $H_{a1}^{\mathrm{eff}}$, $H_{a2}^{\mathrm{eff}}$ and $H_{a3}^{\mathrm{eff}}$ are the equivalent values of $H_{a1}$, $H_{a2}$ and $H_{a3}$, respectively. The formulas for calculating the relevant parameters are as follows:

$r_{c}^{\mathrm{eff}}=(\frac{1-f_{1}}{f_{1}})\frac{D}{2}$ (S47)

$R_{c}^{\mathrm{eff}}=(\frac{1-f_{1a}}{f_{1a}})\frac{d}{2}$ (S48)

$r^{\mathrm{eff}}=(\sqrt{\frac{\pi}{2f_{1}}}-1)\frac{D}{2}$ (S49)

$R^{\mathrm{eff}}=(\sqrt{\frac{\pi}{2f_{1a}}}-1)\frac{d}{2}$ (S50)

$H_{a2}=\frac{\frac{D}{2}[2-3cos\theta_{1}+{cos}^{3}\theta_{1}]}{3{sin}^{3}\theta_{1}}$ (S51)

$H_{a2}^{\mathrm{eff}}=\frac{f_{1}}{1-f_{1}}{(\sqrt{\frac{\pi}{2f_{1}}}-1)}^{3}H_{a2}$ (S52)

$({H_{a3})}_{\max}=\frac{\frac{d}{2}[2-3sin\theta_{\mathrm{adv}}+{sin}^{3}\theta_{\mathrm{adv}}]}{-3{cos}^{3}\theta_{\mathrm{adv}}}$ (S53)

${{(H}_{a3}^{\mathrm{eff}})}_{\max}=\frac{f_{1a}}{1-f_{1a}}{(\sqrt{\frac{\pi}{2f_{1a}}}-1)}^{3}({H_{a3})}_{\max}$ (S54)

Compared to nRS surface, the droplet on RS surface exhibits a higher energy barrier for wetting transition. This barrier can be divided into five components: the energy barrier due to the pinning of TPCL at the top of the structure; the capillary energy barrier associated with the downward movement of TPCL along the sidewall of the cap; the energy barrier caused by the pinning of TPCL at the lower corner of the cap; the capillary energy barrier associated with the downward movement of TPCL along the sidewall of the pillar; and the energy barrier due to the compression of the gas within the structure. In summary, the nondimensionalized total energy barrier for the wetting transition on RS surface can be expressed as:

${\Delta G}_{\mathrm{RS}}^{*}=\left( 1-f_{1} \right)\cdot\left[ \frac{2\mu^{2}}{1+sin\theta_{\mathrm{adv}}}-1-(\frac{2h}{r_{c}^{\mathrm{eff}}}+\frac{2R_{c}^{\mathrm{eff}}}{\left( r_{c}^{\mathrm{eff}} \right)^{2}}(H-h-{{(H}_{m}^{\mathrm{eff}})}_{\max}))\left( f_{2}cos\theta_{Y}+f_{2}-1 \right)+\frac{P_{0}H_{V}^{\mathrm{eff}}}{\gamma_{\mathrm{LV}}}\ln\left( \frac{H_{V}^{\mathrm{eff}}}{H_{V}^{\mathrm{eff}}-h-\mu^{2}({{(H}_{a3}^{\mathrm{eff}})}_{\max}+H-h-{{(H}_{m}^{\mathrm{eff}})}_{\max})} \right) \right]$ (S55)

Due to the significant enhancement of the energy barrier in the wetting process by the reentrant angle feature, we separately calculate the energy barrier when the TPCL moves to the lower corner of RS cap. When $h_{1}=h$ and $\theta_{1}=\frac{\pi}{2}$, TPCL becomes pinned at the lower corner of RS cap, and the energy barrier at this point can be expressed as:

${\Delta G}_{\mathrm{cap}}^{*}=\left( 1-f_{1} \right)\cdot\left[ 1-\frac{2h}{r_{c}^{\mathrm{eff}}}\left( f_{2}cos\theta_{Y}+f_{2}-1 \right)+\frac{P_{0}H_{V}^{\mathrm{eff}}}{\gamma_{\mathrm{LV}}}\ln\left( \frac{H_{V}^{\mathrm{eff}}}{H_{V}^{\mathrm{eff}}-H_{a2}^{\mathrm{eff}}-h} \right) \right]$ (S56)

Here, ${\Delta G}_{\mathrm{cap}}^{*}$ represents the nondimensionalized cap barrier. For RS surface, $h=30 \mu m$, $H=200 \mu m$, $D=85 \mu m$, $d=45 \mu m$, $f_{1}=0.175$, $\theta_{2}\approx\frac{\pi}{4}$ and $H_{1}\approx78 \mu m$. In this study, the initial pressure $P_{0}$ is set to standard atmospheric pressure, $P_{0}=101.325 \mathrm{kPa}$. Additionally, $r_{0}=15 \mu m$, $f_{2}=0.3$ and $\gamma_{\mathrm{LV}}=72.8 mN m^{-1}$.

# Supplementary Table

**Table S1.** Detailed fabrication processes for different structural surfaces

| Types | Processing | Laser Fluence (J cm^-2^) | Scanning Speed (mm s^-1^) | | Repetition frequency | | | | Pulse Duration | | Scanning Route | | Scanning Pitch ($\mu m$) |
| --- | --- | --- | --- | --- | --- | --- | --- | --- | --- | --- | --- | --- | --- |
| nRS | Laser ablation | 0.5-1.6 | 2000-3000 | | 300 kHz | | | 150 ns | | | Crossed lines | | 10 |
|  | Wet Chemical Reaction | Solution: 2.5 mol L^-1^ NaOH +  0.1 mol L^-1^ (NH_4_)_2_S_2_O_8_  Temperature: 10-30 ℃  Time: 20-50 min | | | | | | | | | | | |
| RS | Laser ablation | 0.5-1.6 | | 2000-3000 | | 300 kHz | 150 ns | | | Crossed lines | | 10 | |
|  | Hot pressing | Force: 60-120 N  Temperature: 500-800 ℃ | | | | | | | | | | | |
|  | Wet Chemical Reaction | Solution: 2.5 mol L^-1^ NaOH +  0.1 mol L^-1^ (NH_4_)_2_S_2_O_8_  Temperature: 10-30 ℃  Time: 20-50 min | | | | | | | | | | | |

# Supplementary Figures

**
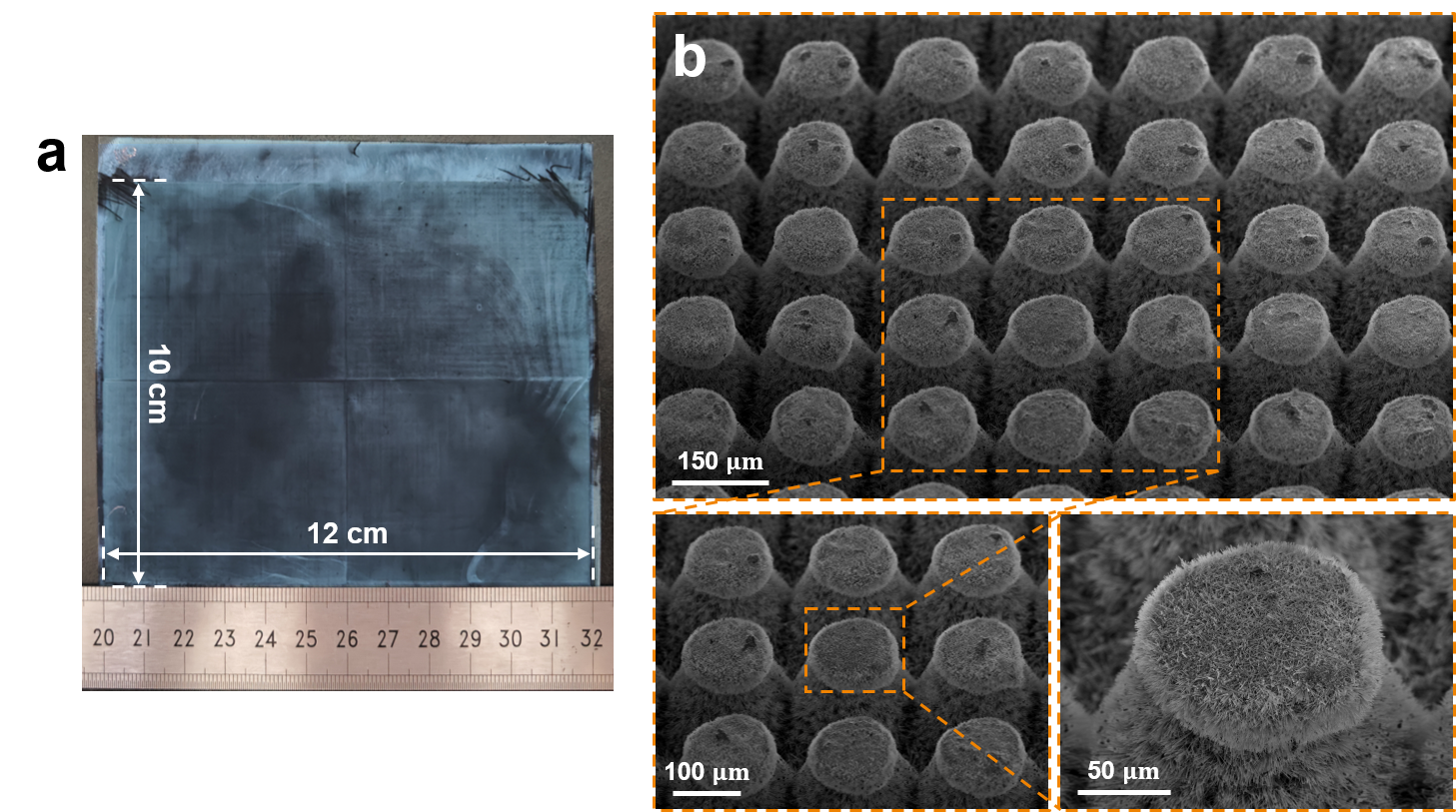
**

**Figure S1. Large-area structure fabricated on copper substrate. (a)** Macroscopic photograph. **(b)** SEM images of micro-nanostructures.

**
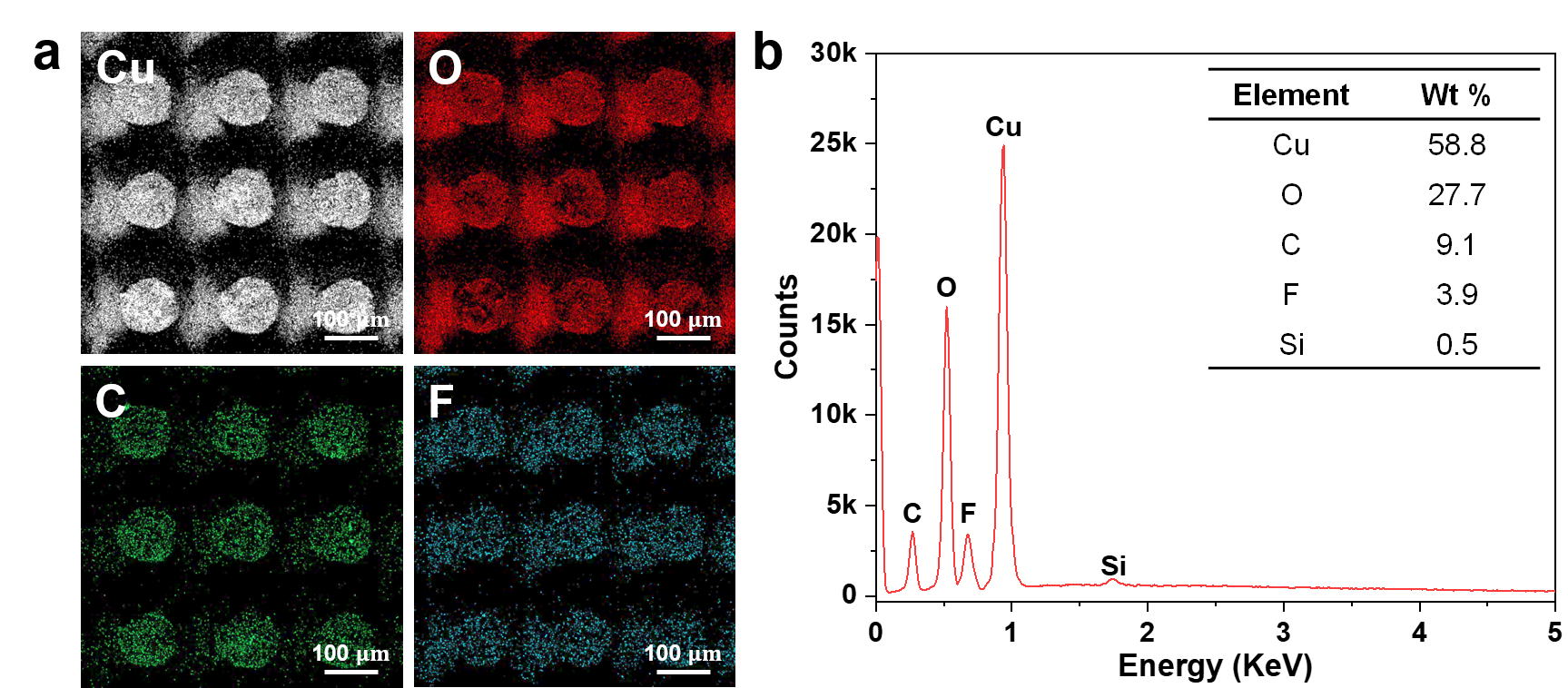
**

**Figure S2. The initial state of RS surface. (a)** Distribution of different elements on RS surface. **(b)** Mass percentages of the different elements.

**Figure S3. The room-temperature superhydrophobicity of the two surfaces.** Data are the mean ± s.d. of at least three independent measurements.

**Figure S4. The contact angles and sliding angles of various liquids on RS surface.** The inset shows optical images of different droplets on RS surface. Data are the mean ± s.d. of at least three independent measurements.

**Figure S5. Schematic illustration of pressure-induced wetting transitions on nRS and RS surfaces.** The geometric parameters of the structures are marked with symbols.

**Figure S6. The relationship between contact angles, ice adhesion strength, and the pillar spacing, pillar height, and cap diameter of RS.** Data are the mean ± s.d. of at least three independent measurements.

**
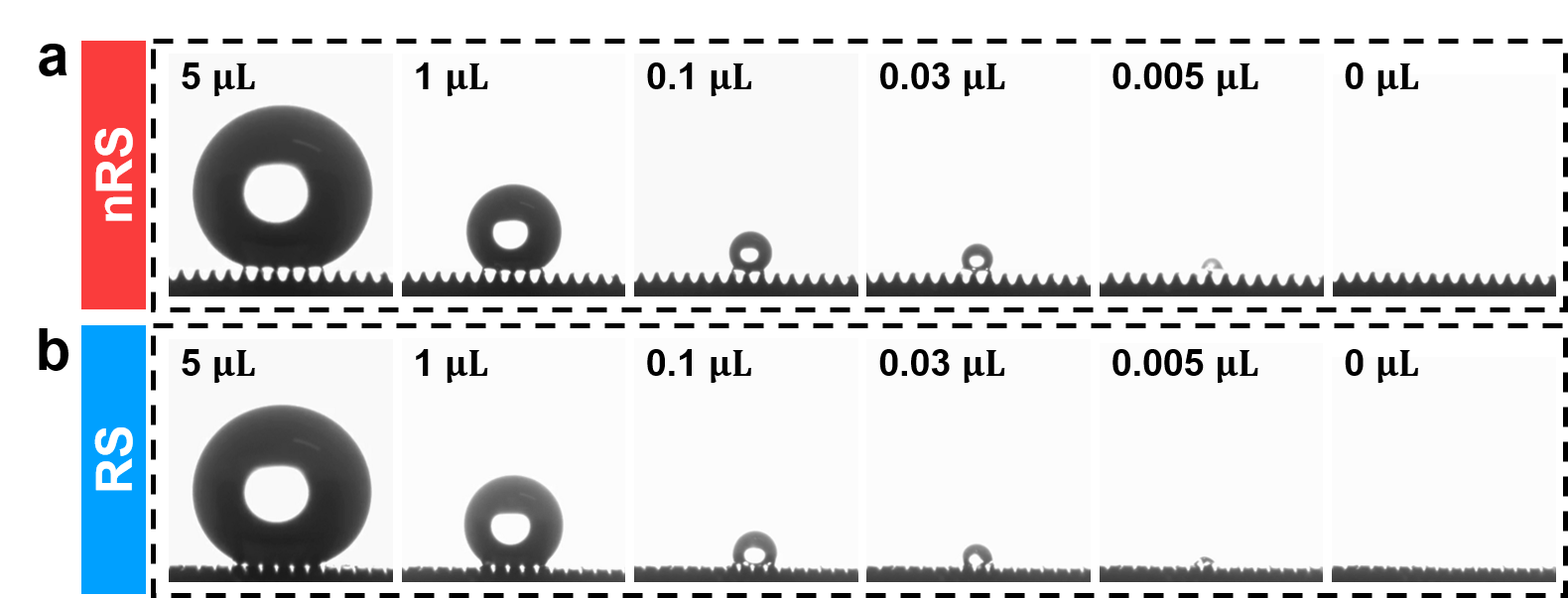
**

**Figure S7. Side-view optical images of the droplet evaporation process on the two surfaces.**

**Figure S8. The pressure resistance of the two surfaces in maintaining the CB state at room temperature. (a)** and **(b)** show the variations in the contact angle and TPCL diameter of the droplet with Laplace pressure, respectively. **(c)** and **(d)** represent the Laplace pressures derived from the contact angles and TPCL diameters for the two surfaces.

**Figure S9. Comparison of the critical Laplace pressure between our fabricated surfaces and other reported surfaces.** Other reported data are obtained from references.^[3–16]^

**Figure S10. Schematic illustration of the experimental setup for recording droplet freezing behavior.**

**
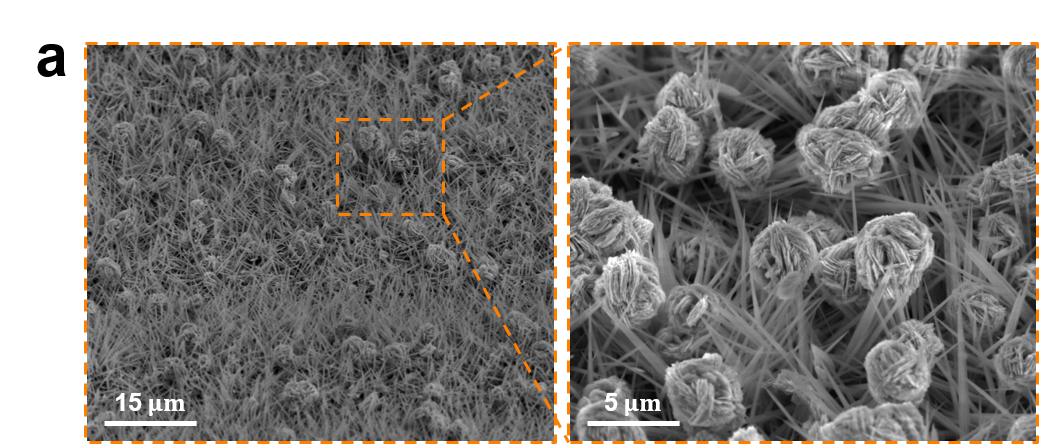
**

**Figure S11. NWs directly prepared on flat surfaces. (a)** SEM images of the NWs. **(b)** Icing delay performances of the RS versus the NW surfaces at various low temperatures. Data between -45 °C and -33 °C are shown in the enlarged box. Humidity was controlled at 70 ± 5% RH.

**
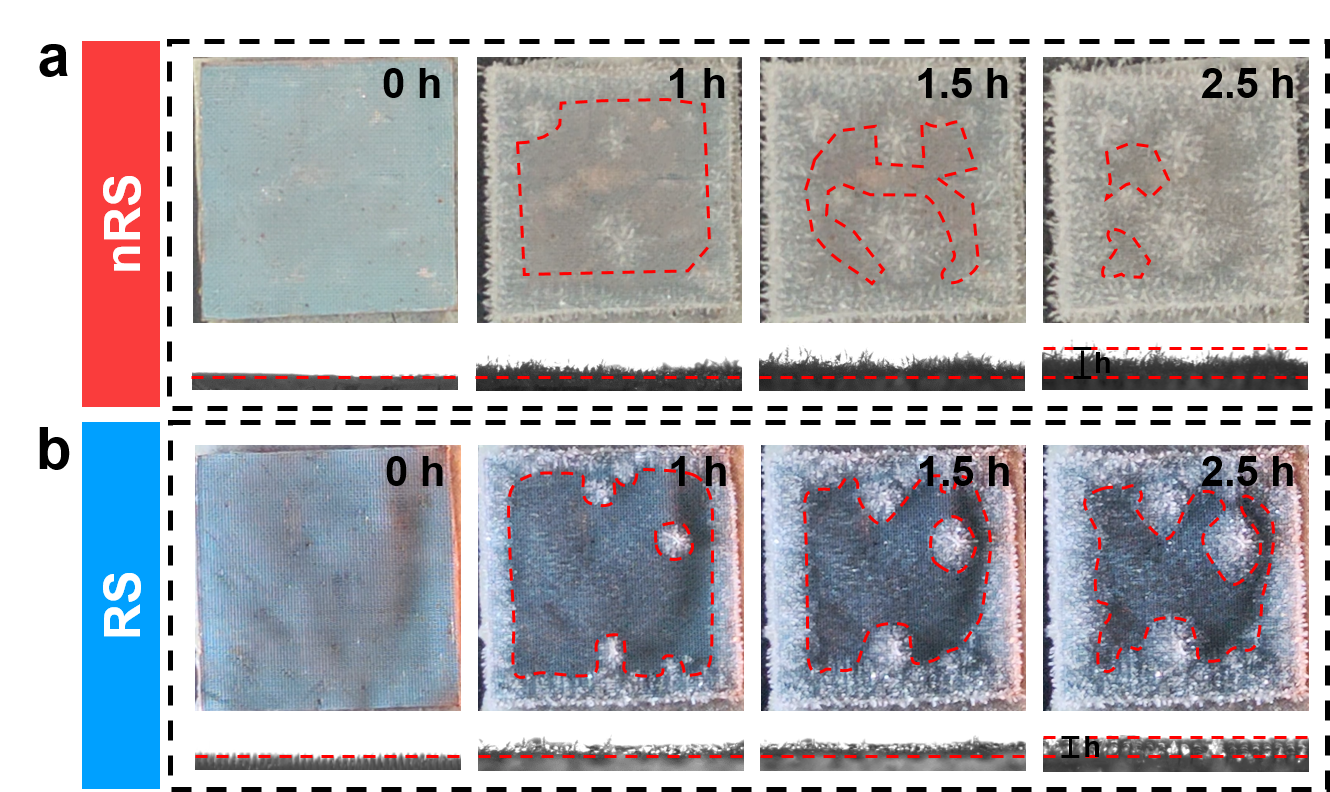
**

**Figure S12. Top-view and side-view images of the static frosting process on the two surfaces. (a)** nRS surface. **(b)** RS surface.

**Figure S13. Thresholds for the anti-icing performance of RS surface. (a)** Delay icing time under different temperatures and humidities. **(b)** Range of environmental conditions where the anti-icing performance is effective.

**Figure S14. The relationship between air pocket pressure and temperature for the two surfaces.**

**
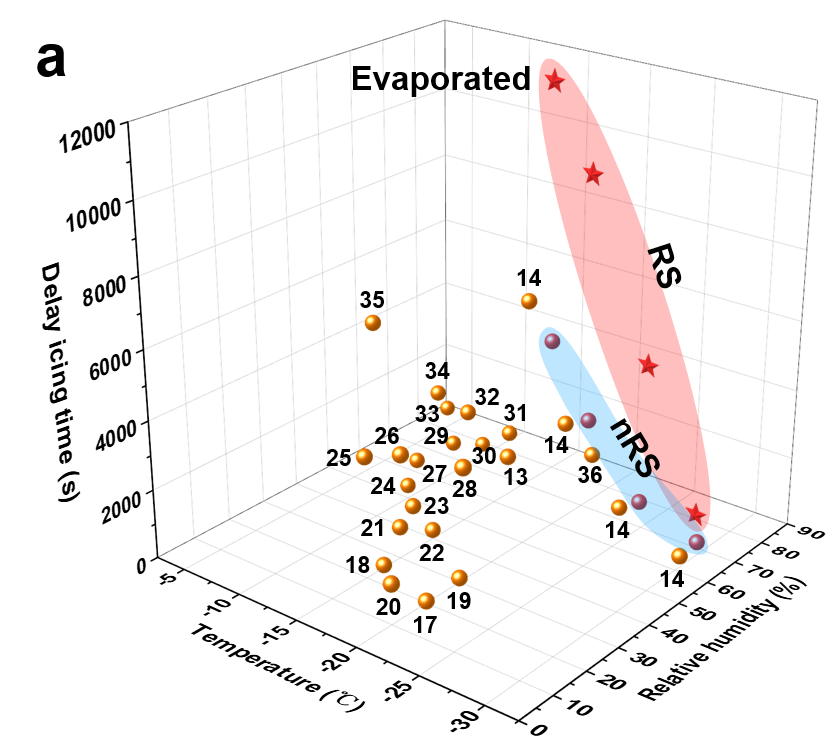
**

**Figure S15. Comparison of delay icing time between the two surfaces and anti-icing surfaces reported in other studies under different environmental conditions.** Other reported data are obtained from references.^[13,14,17–36]^

**
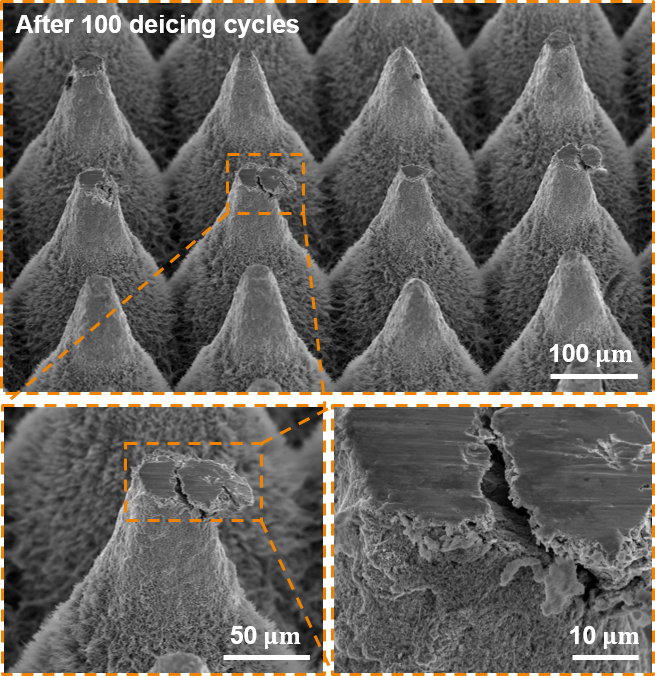
**

**Figure S16. Microstructural images of the nRS surface after 100 deicing cycles.** The NWs in the upper region of the nRS surface completely disappeared, with wear damage at the tops of some microcones. This hierarchical degradation of micro-nanostructures accounts for the significant deterioration in icephobicity.

**
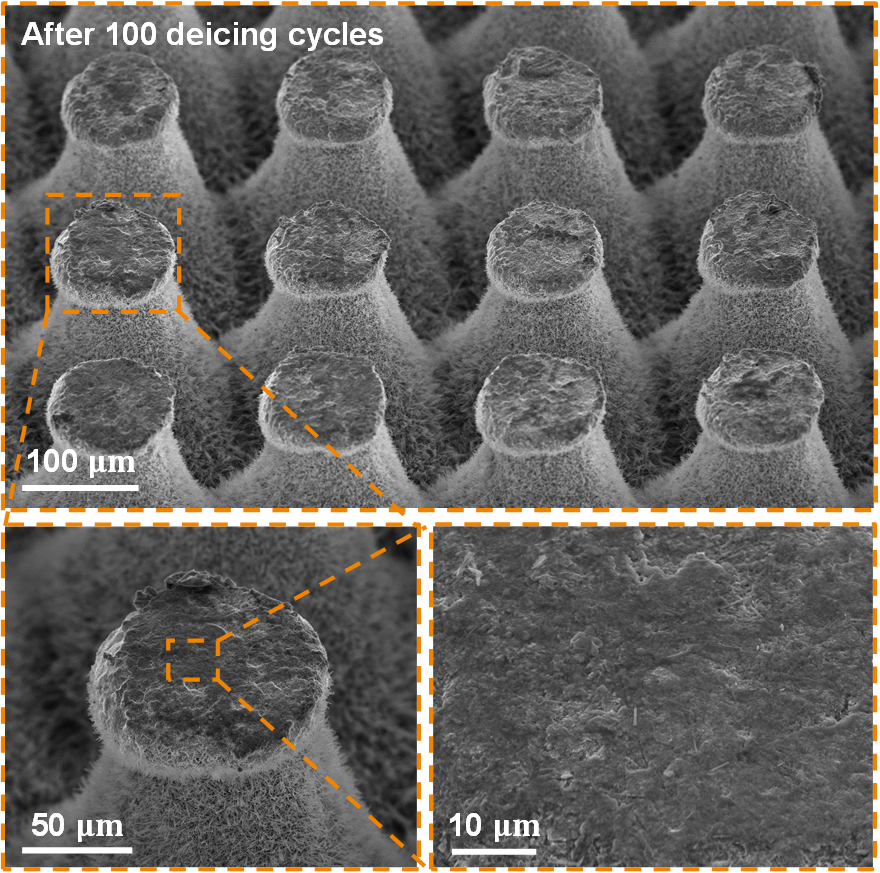
**

**Figure S17. SEM images of the micro-nanostructures on RS surface after 100 icing and deicing cycles.** Multiple deicing cycles only damaged the nanostructures at the top of the microstructures, while the microstructures and the nanostructures on the sidewalls and bottom remained well.

**
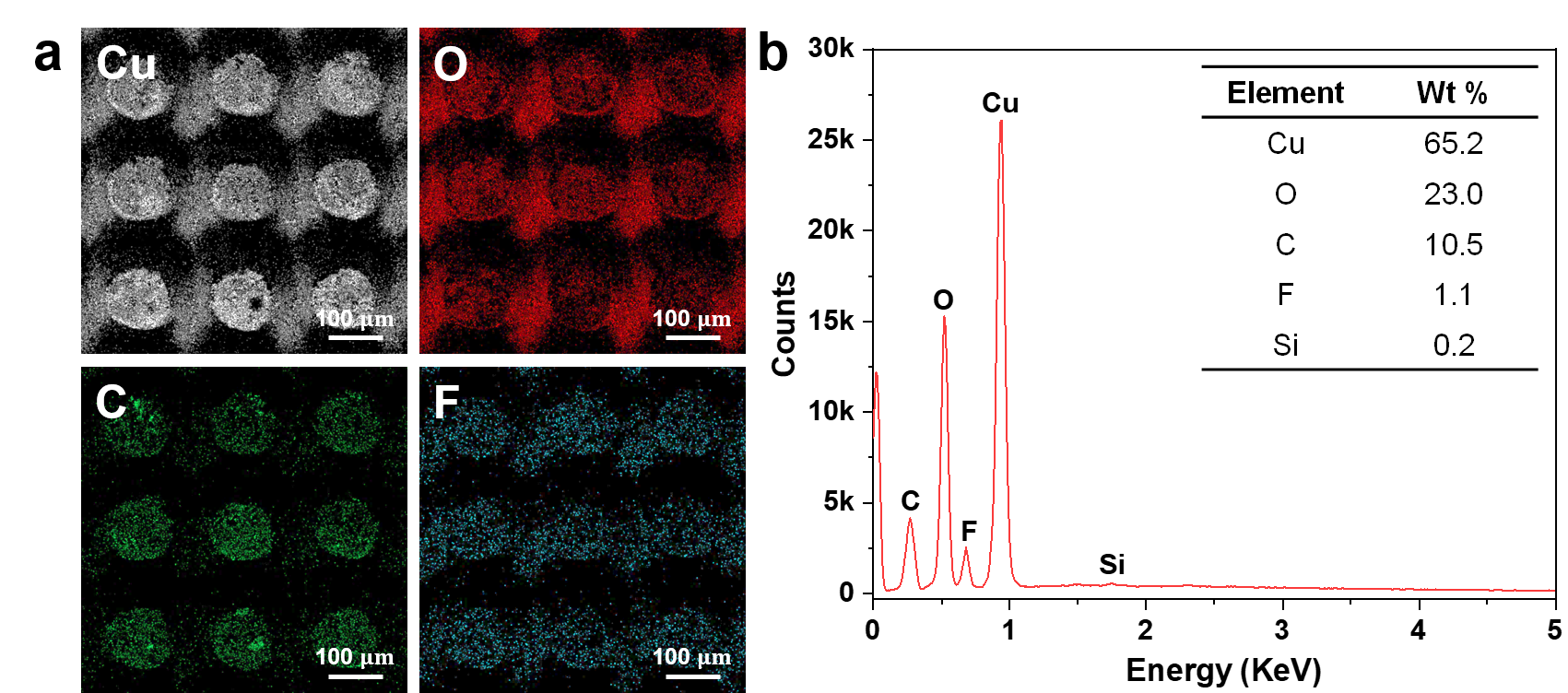
**

**Figure S18. EDS images and elemental mass ratios of RS surface after 100 icing and deicing cycles.** The content of low surface energy elements F and Si decreased, which compromised the surface's icephobicity.

**Figure S19. Thresholds for the deicing performance of RS surface.** **(a)** Ice adhesion strength under different temperatures and humidities. **(b)** Range of environmental conditions where the deicing performance is effective.

**Figure S20. Comparison of ice adhesion strength and contact angles between the two surfaces and those reported in other studies.** Other reported data are obtained from references.^[3,13,14,17,18,20,29,32,33,36–48]^

**Figure S21. The ice adhesion strength of our fabricated surfaces after multiple deicing cycles was compared with that of other reported surfaces.** Other reported data are obtained from references.^[3,13,14,17,18,20,29,32,33,36–42,44–47,49,50]^

**
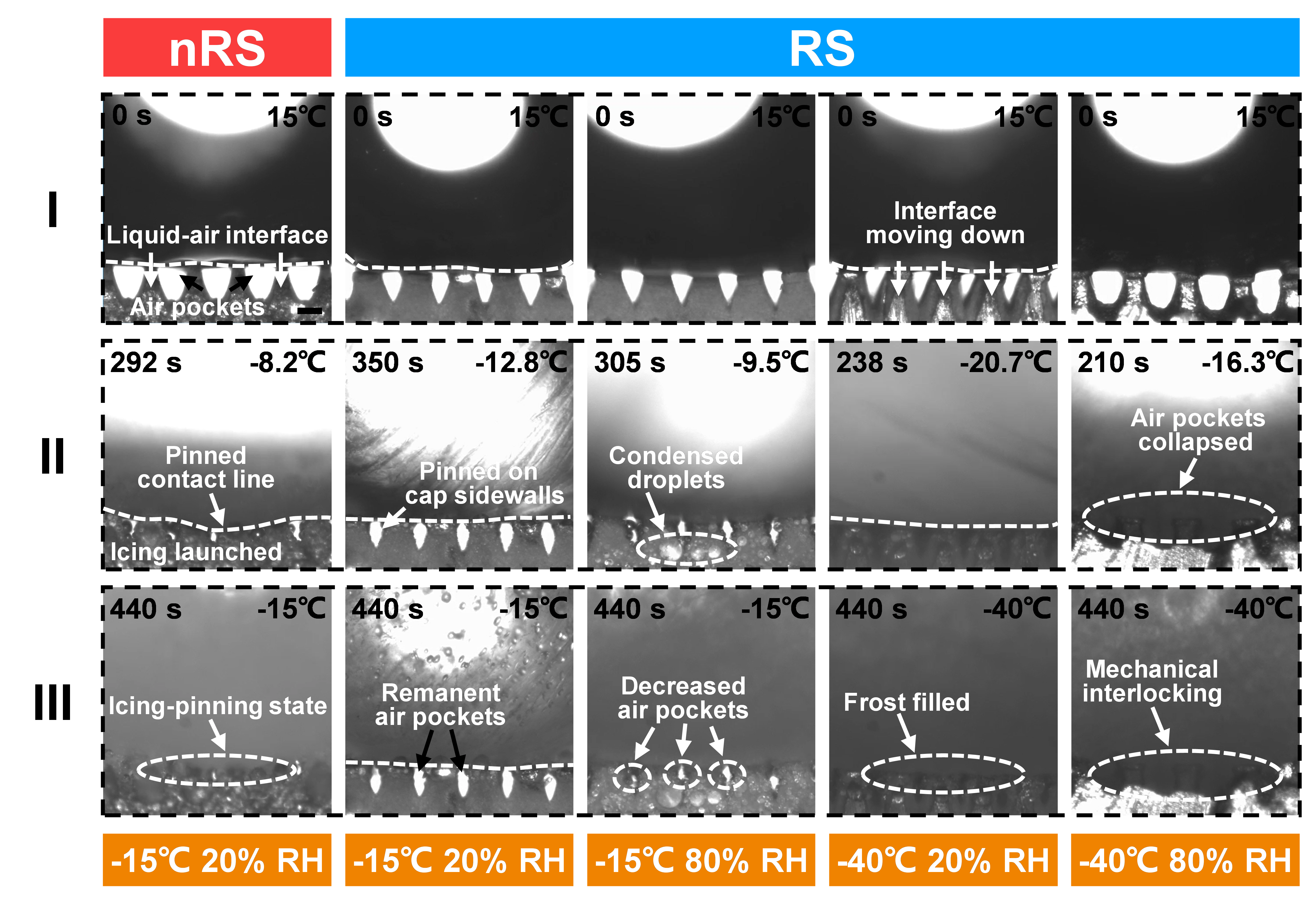
**

**Figure S22. Side-view observations of triple-phase interface evolution on nRS and RS surfaces under varying environmental conditions.** The scale bar is 100 μm.

**Figure S23.** **The PPD and RAPR of droplets on nRS and RS surfaces after freezing completion (-15 °C and 20% RH).** The insets illustrate the geometric meanings of each parameter. The pinning depth percentage (PPD) represents the depth $h_{1}$ to which the droplet is pinned after freezing as a percentage of the initial air pocket height *H*, $\delta_{p}=\frac{h_{1}}{H}\times100\%$. The remaining air pocket volume ratio (RAPR) represents the ratio of the remaining air pocket volume $V_{r}$ after freezing to the initial air pocket volume $V_{o}$, $\delta_{r}=\frac{V_{r}}{V_{o}}\times100\%$.

**Figure S24. The PPD and RAPR of droplets on RS surface after freezing under different environmental conditions.**

**Figure S25. Schematic illustration of the droplet wetting process on RS and nRS surfaces.**

**Figure S26. The nondimensionalized total energy barriers and the energy barriers at the caps for the wetting transition on the two surfaces.**

**Figure S27. Variation in contact diameter with temperature during the icing and melting processes on the two surfaces.** **(a)** nRS surface. **(b)** RS surface.

**Figure S28. Variation in contact angle and contact diameter of droplets with temperature on the two surfaces.** **(a)** Contact angle. **(b)** Contact diameter.

**Figure S29. The effects of different structures and the number of icing-melting cycles on the CARD and CDRD of droplets on the surfaces. (a)** CARD and CDRD of droplets on the two surfaces after icing and melting. **(b)** CARD and CDRD of droplets on RS surface after multiple icing and melting cycles. The insets illustrate the calculation principles. The contact angle retention degree (CARD) is the percentage of the contact angle $\theta_{a}$ after icing-melting cycles relative to the initial contact angle $\theta_{b}$, $\delta_{\mathrm{CA}}=\frac{\theta_{a}}{\theta_{b}}\times100\%$. The contact diameter retention degree (CDRD) is the percentage of the initial contact diameter $D_{b}$ relative to the contact diameter $D_{a}$ after icing-melting cycles, $\delta_{\mathrm{CD}}=\frac{D_{b}}{D_{a}}\times100\%$.

**Figure S30. A comprehensive comparison of the room-temperature and low-temperature performance between nRS and RS surfaces.**

**
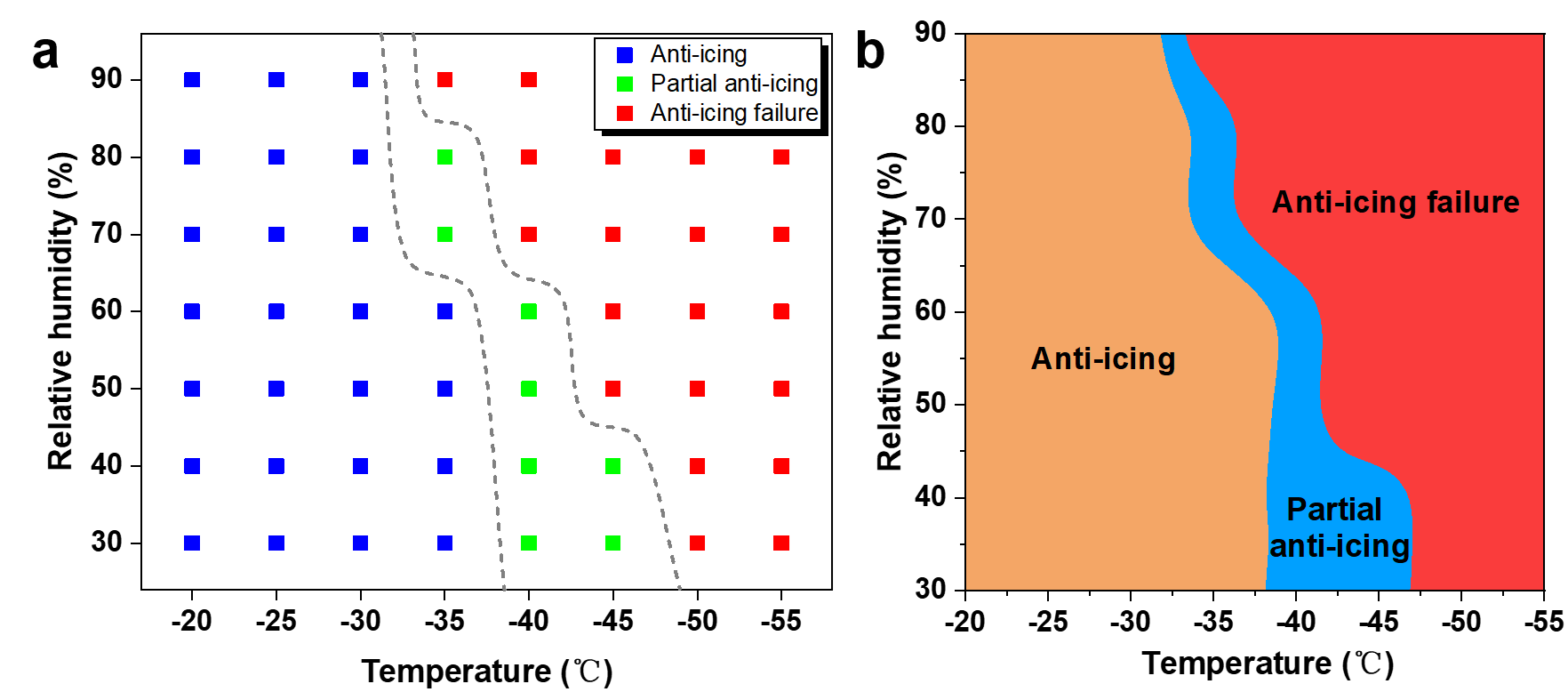
**

**Figure S31. Thresholds for the anti-icing/deicing performance of RS surface.** **(a)** Anti-icing/deicing performance under different temperatures and humidities. **(b)** Range of environmental conditions where the anti-icing/deicing performance is effective.

**
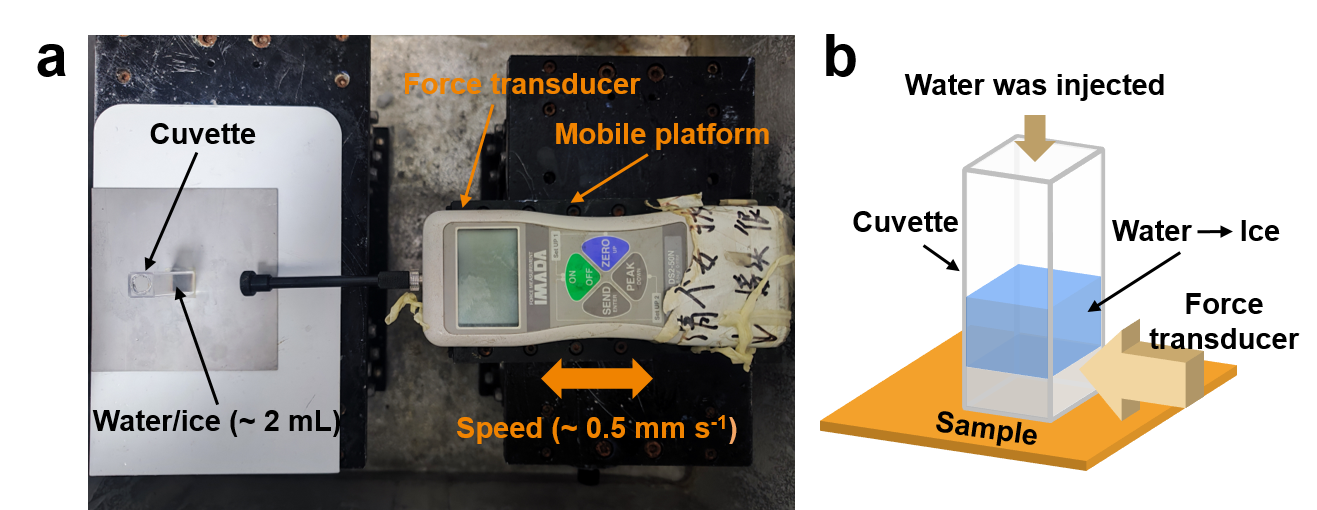
**

**Figure S32. Photo and schematic of the experimental setup used for measuring ice adhesion strength.** **(a)** Photograph. **(b)** Schematic illustration.

# Supplementary Videos

**Video S1**

Ice-pinning process of droplet on nRS surface (-15 ℃ and 20% RH).

**Video S2**

Icing process of droplet on RS surface (-15 °C and 20% RH, -40 °C and 80% RH).

**Video S3**

Icing and melting cycle of droplet on nRS surface.

**Video S4**

Icing and melting cycle of droplet on RS surface.

**References**

[1] Y. Xue, S. Chu, P. Lv, H. Duan, *Langmuir* **2012**, *28*, 9440.

[2] H. Wu, Z. Yang, B. Cao, Z. Zhang, K. Zhu, B. Wu, S. Jiang, G. Chai, *Langmuir* **2017**, *33*, 407.

[3] R. Pan, H. Zhang, M. Zhong, *ACS Appl. Mater. Interfaces* **2021**, *13*, 1743.

[4] X. Chen, J. A. Weibel, S. V. Garimella, *Sci Rep* **2015**, *5*, 17110.

[5] P. Tsai, R. G. H. Lammertink, M. Wessling, D. Lohse, *Phys. Rev. Lett.* **2010**, *104*, 116102.

[6] X. Yan, Y. Qin, F. Chen, G. Zhao, S. Sett, M. J. Hoque, K. F. Rabbi, X. Zhang, Z. Wang, L. Li, F. Chen, J. Feng, N. Miljkovic, *ACS Nano* **2020**, *14*, 12796.

[7] J. Long, L. Pan, P. Fan, D. Gong, D. Jiang, H. Zhang, L. Li, M. Zhong, *Langmuir* **2016**, *32*, 1065.

[8] C. Liu, M. Zhao, D. Lu, Y. Sun, L. Song, Y. Zheng, *Langmuir* **2022**, *38*, 6923.

[9] T. Cai, Z. Jia, H. Yang, G. Wang, *Colloid Polym Sci* **2016**, *294*, 833.

[10] D. Liu, R. Liu, L. Cao, L. Wang, S. Saeed, Z. Wang, P. Bryanston-Cross, *Langmuir* **2024**, *40*, 950.

[11] Superhydrophobicity and compressive stability of Salvinia surface: Mechanical analysis and structural design, *Applied Surface Science* **2023**, *627*, 157274.

[12] P. Sun, Y. Jin, Y. Yin, C. Wu, C. Song, Y. Feng, P. Zhou, X. Qin, Y. Niu, Q. Liu, J. Zhang, Z. Wang, X. Hao, *Small Methods* **2024**, *8*, 2201602.

[13] S. Xuan, H. Yin, G. Li, Z. Zhang, Y. Jiao, Z. Liao, J. Li, S. Liu, Y. Wang, C. Tang, W. Wu, G. Li, K. Yin, *ACS Nano* **2023**, *17*, 21749.

[14] L. Wang, D. Li, G. Jiang, X. Hu, R. Peng, Z. Song, H. Zhang, P. Fan, M. Zhong, *ACS Nano* **2024**, *18*, 12489.

[15] L. Hu, W. Wang, A. Pan, W. Duan, X. Mei, M. Zhou, *Optics & Laser Technology* **2025**, *181*, 111565.

[16] Z. Chen, L. Song, Y. Wang, H. Tao, Z. Liu, T. Wang, F. Ye, Y. He, J. Lin, *Applied Surface Science* **2024**, *655*, 159454.

[17] C. Chen, Z. Tian, X. Luo, G. Jiang, X. Hu, L. Wang, R. Peng, H. Zhang, M. Zhong, *Chemical Engineering Journal* **2022**, *450*, 137936.

[18] L. Wang, G. Jiang, Z. Tian, C. Chen, X. Hu, R. Peng, H. Zhang, P. Fan, M. Zhong, *Mater. Horiz.* **2023**, *10*, 209.

[19] F. Zhang, H. Yan, M. Chen, *Small* **2024**, *20*, 2312226.

[20] X. Fu, Y. Shen, A. Yeerken, J. Jiang, Y. Lin, Z. Wang, Y. Xu, J. Tao, *Chemical Engineering Journal* **2024**, *497*, 154240.

[21] M. Zhou, L. Zhang, L. Zhong, M. Chen, L. Zhu, T. Zhang, X. Han, Y. Hou, Y. Zheng, *Advanced Materials* **2024**, *36*, 2305322.

[22] M. Wu, Y. Liu, Z. Zhang, Y. Wang, H. He, H. Zhu, K. Xu, J. Wang, J. Lu, *Surface and Coatings Technology* **2024**, *483*, 130820.

[23] S. K. Sharma, R. Pradhan, H. S. Grewal, *ACS Appl. Mater. Interfaces* **2024**, *16*, 44139.

[24] J. Li, W. Jiao, Y. Wang, Y. Yin, X. He, *Chemical Engineering Journal* **2022**, *434*, 134710.

[25] Z. Xie, H. Wang, Y. Geng, M. Li, Q. Deng, Y. Tian, R. Chen, X. Zhu, Q. Liao, *ACS Appl. Mater. Interfaces* **2021**, *13*, 48308.

[26] L. Zhang, B. Luo, K. Fu, C. Gao, X. Han, M. Zhou, T. Zhang, L. Zhong, Y. Hou, Y. Zheng, *Advanced Science* **2023**, *10*, 2304187.

[27] J. Chu, X. Feng, Y. Li, F. Li, G. Tian, *Langmuir* **2024**, *40*, 10313.

[28] W. Xing, Z. Li, H. Yang, X. Li, X. Wang, N. Li, *Materials & Design* **2019**, *183*, 108156.

[29] X. Feng, J. Chu, G. Tian, Z. Wang, W. Zhou, X. Zhang, Z. Lian, *ACS Appl. Mater. Interfaces* **2023**, *15*, 53159.

[30] A. Gaddam, H. Sharma, T. Karkantonis, S. Dimov, *Applied Surface Science* **2021**, *552*, 149443.

[31] Y. Wu, X. Shu, Y. Yang, W. She, L. Dong, Q. Ran, *Chemical Engineering Journal* **2023**, *463*, 142444.

[32] L. Lai, W. Wang, Y. Yang, G. Li, S. Liu, K. Yin, *Surfaces and Interfaces* **2024**, *51*, 104679.

[33] Y. Shu, X. Lu, W. Lu, W. Su, Y. Wu, H. Wei, D. Xu, J. Liang, Y. Xie, *Surface and Coatings Technology* **2023**, *455*, 129216.

[34] A. Xia, L. He, S. Qie, J. Zhang, H. Li, N. He, X. Hao, *Applied Sciences* **2022**, *12*, 2119.

[35] Y. Qi, Z. Yang, W. Huang, J. Zhang, *Applied Surface Science* **2021**, *538*, 148131.

[36] S. Xuan, L. Zhuo, G. Li, Q. Zeng, J. Liu, J. Yu, L. Chen, Y. Yang, S. Liu, Y. Wang, K. Yin, *Small* *n/a*, 2404979.

[37] Y. Lei, B. Jiang, H. Liu, F. Zhang, Y. An, Y. Zhang, Y. Yuan, J. Xu, X. Li, T. Liu, *Progress in Organic Coatings* **2023**, *183*, 107795.

[38] S. M. Mirmohammadi, H. D. Shirazi, M. Heikkilä, S. Franssila, J. Vapaavuori, V. Jokinen, *Small* **2024**, *20*, 2403863.

[39] S. Xuan, H. Yin, G. Li, Y. Yang, Y. Wang, J. Liu, S. Liu, X. Li, Y. Song, T. Wu, K. Yin, *Mater. Horiz.* **2024**, *11*, 3561.

[40] P. Wang, H. Zhao, B. Zheng, X. Guan, B. Sun, Y. Liao, Y. Yue, W. Duan, H. Ding, *J Bionic Eng* **2023**, *20*, 1891.

[41] Z. Tian, L. Wang, D. Zhu, C. Chen, H. Zhao, R. Peng, H. Zhang, P. Fan, M. Zhong, *ACS Appl. Mater. Interfaces* **2023**, *15*, 6013.

[42] Y. Hou, K. L. Choy, *Progress in Organic Coatings* **2022**, *163*, 106637.

[43] J. Chu, X. Feng, Y. Li, F. Li, G. Tian, *Langmuir* **2024**, *40*, 10313.

[44] Q. He, Y. Jia, H. Wang, J. He, J. Wang, Y. Xu, Y. Liu, A. Li, *Materials & Design* **2024**, *237*, 112516.

[45] Y. Wu, X. Shu, Y. Yang, W. She, L. Dong, Q. Ran, *Chemical Engineering Journal* **2023**, *463*, 142444.

[46] Y. Zhao, Y. Liu, Q. Liu, W. Guo, L. Yang, D. Ge, *Materials Letters* **2018**, *233*, 263.

[47] C. Chen, Z. Tian, X. Luo, G. Jiang, X. Hu, L. Wang, R. Peng, H. Zhang, M. Zhong, *ACS Appl. Mater. Interfaces* **2022**, *14*, 23973.

[48] J. Li, W. Jiao, Y. Wang, Y. Yin, X. He, *Chemical Engineering Journal* **2022**, *434*, 134710.

[49] Z. Tian, P. Fan, D. Zhu, L. Wang, H. Zhao, C. Chen, R. Peng, D. Li, H. Zhang, M. Zhong, *Chemical Engineering Journal* **2023**, *473*, 145382.

[50] W. Pan, Q. Wang, J. Ma, W. Xu, J. Sun, X. Liu, J. Song, *Advanced Functional Materials* **2023**, *33*, 2302311.
